# Lipidation of Pneumococcal Antigens Leads to Improved Immunogenicity and Protection

**DOI:** 10.3390/vaccines8020310

**Published:** 2020-06-17

**Authors:** Franziska Voß, Lucille F. van Beek, Dominik Schwudke, Thomas H. A. Ederveen, Fred J. van Opzeeland, Daniela Thalheim, Sidney Werner, Marien I. de Jonge, Sven Hammerschmidt

**Affiliations:** 1 Department of Molecular Genetics and Infection Biology, Interfaculty Institute of Genetics and Functional Genomics, Center for Functional Genomics of Microbes, University of Greifswald, 17489 Greifswald, Germany; franziska.voss@uni-greifswald.de (F.V.); da.thalheim@googlemail.com (D.T.); sag92@gmx.de (S.W.); 2 Section Pediatric Infectious Diseases, Laboratory of Medical Immunology, Radboud Institute for Molecular Life Sciences, Radboudumc, 6525 GA Nijmegen, The Netherlands; lucille.vanbeek@radboudumc.nl (L.F.v.B.); fred.vanopzeeland@radboudumc.nl (F.J.v.O.); marien.dejonge@radboudumc.nl (M.I.d.J.); 3 Radboud Center for Infectious Diseases, Radboudumc, 6525 GA Nijmegen, The Netherlands; 4 Division of Bioanalytical Chemistry, Priority Area Infection, Research Center Borstel, Leibniz Center for Medicine and Bioscience, 23845 Borstel, Germany; dschwudke@fz-borstel.de; 5 German Center for Infection Research (DZIF), 38124 Braunschweig, Germany; 6 Airway Research Center North Member of the German Center for Lung Research (DZL), 22927 Großhansdorf, Germany; 7 Center for Molecular and Biomolecular Informatics, Radboud Institute for Molecular Life Sciences, Radboud University Medical Center, 6525 GA Nijmegen, The Netherlands; tom.ederveen@radboudumc.nl

**Keywords:** *Streptococcus pneumoniae*, lipoproteins, lipidation, pneumococcal colonization, vaccine, protection, immune response

## Abstract

*Streptococcus pneumoniae* infections lead to high morbidity and mortality rates worldwide. Pneumococcal polysaccharide conjugate vaccines significantly reduce the burden of disease but have a limited range of protection, which encourages the development of a broadly protective protein-based alternative. We and others have shown that immunization with pneumococcal lipoproteins that lack the lipid anchor protects against colonization. Since immunity against *S. pneumoniae* is mediated through Toll-like receptor 2 signaling induced by lipidated proteins, we investigated the effects of a lipid modification on the induced immune responses in either intranasally or subcutaneously vaccinated mice. Here, we demonstrate that lipidation of recombinant lipoproteins DacB and PnrA strongly improves their immunogenicity. Mice immunized with lipidated proteins showed enhanced antibody concentrations and different induction kinetics. The induced humoral immune response was modulated by lipidation, indicated by increased IgG2/IgG1 subclass ratios related to Th1-type immunity. In a mouse model of colonization, immunization with lipidated antigens led to a moderate but consistent reduction of pneumococcal colonization as compared to the non-lipidated proteins, indicating that protein lipidation can improve the protective capacity of the coupled antigen. Thus, protein lipidation represents a promising approach for the development of a serotype-independent pneumococcal vaccine.

## 1. Introduction

Despite the implementation of pneumococcal polysaccharide conjugate vaccines (PCV), *Streptococcus pneumoniae* (pneumococcus) is still a major cause of morbidity and mortality worldwide, especially in young children, the elderly, and immune-compromised individuals [1,2]. The major disadvantages of PCVs include the high manufacturing costs and limited serotype coverage, which facilitates the replacement of vaccine serotypes by non-vaccine serotypes, hence requiring alternative immunization strategies in the near future [3,4].

Because of these shortcomings, efforts have been made to develop novel vaccines based on broadly representative, serotype-independent, highly conserved pneumococcal protein antigens. Pneumococcal lipoproteins might be promising candidates for a future protein-based vaccine as they represent the largest group of surface-exposed and conserved proteins of *S. pneumoniae* and contribute to pneumococcal pathogenesis [5,6,7]. Indeed, various pneumococcal lipoproteins have been shown to protect against pneumococcal infection in *in vivo* models [6,8,9].

Importantly, recognition of *S. pneumoniae* by the host immune system is characterized by inflammation initiated through interactions between bacterial ligands and host cell surface receptors. Among these, Toll-like receptors such as TLR2 play a fundamental role [10]. TLR2 has been shown to be essential for clearance of *S. pneumoniae* in mouse colonization, meningitis, and otitis media models [11,12,13,14,15]. Moreover, the generation of adaptive humoral and cellular immune responses to *S. pneumoniae* is driven by TLR2 signaling, which has been shown to be involved in shaping immune responses related to Th1-type immunity [15,16,17].

Thus, pneumococcal ligands stimulating TLR2 are important for the establishment of a potent immune response. One of those ligands is the lipid moiety at the N-terminus of mature lipoproteins, which enables embedding of these proteins into the cytoplasmic membrane [18]. In previous studies, the immune-stimulating capacity of lipoproteins has been demonstrated and was shown to provide protection against pneumococcal colonization. Vaccination of mice with lipidated proteins MalX and GshT reduced the bacterial load in nasal washes compared to non-lipidated proteins, an effect that was abrogated in TLR2-deficient mice [19]. In addition, lipidation and surface-localization of lipoproteins were shown to be critical for the immunogenicity and protective capacity of pneumococcal whole cell vaccines [20]. Importantly, protection against colonization was associated with increased Interleukin (IL) 17A responses that were dependent on lipoprotein-driven activation of TLR2 [19,20]. In addition to IL-17A responses, antibody-mediated mechanisms have been shown to be crucial for containment of pneumococcal colonization and subsequent lung infection [21,22,23]. With regard to lipidated pneumococcal antigens, however, no detailed analyses of the humoral immune response have been performed so far. Furthermore, it remains to be elucidated to what extent these responses have an impact on the protection against pneumococcal colonization.

In the present study, we provide a detailed analysis of the lipidation-associated effects of pneumococcal lipoproteins on the mouse immune response and the protective capacity of these lipidated antigens. Two lipoproteins have been selected, l,d-carboxypeptidase DacB and the nucleoside-binding protein PnrA, which have previously been shown to be involved in pneumococcal virulence and to protect against pneumococcal colonization when used in the non-lipidated form [5,7,9]. Lipidated DacB or PnrA were used in either intranasal or subcutaneous vaccinations to elucidate the impact of the immunization route on the induced humoral immune response and protection. In addition, the impact of adjuvantation was addressed in this study to evaluate whether the use of an adjuvant has a beneficial effect on the immune response and protectivity of the model antigens used in this study.

We determined that antigen lipidation strongly influences the antibody induction kinetics and leads to increased mucosal and systemic antibody levels. Moreover, lipidation modulates the induced humoral immune response indicated by an increased IgG2/IgG1 subclass ratio related to Th1-type immunity. However, local and systemic cytokine responses and thus cellular immune responses are not strongly affected by protein lipidation. Following intranasal pneumococcal challenge, lipidation mildly improves the protective capacity of the antigens against colonization. We show that, in addition to IL-17A, protection correlated with the elevated antibody levels induced by protein lipidation. Therefore, lipidation of antigens is a promising strategy for the development of a serotype-independent pneumococcal vaccine that would reduce pneumococcal carriage.

## 2. Materials and Methods

### 2.1. Cloning and Purification of Recombinant Lipidated and Non-Lipidated Proteins

For the generation of heterologous expression constructs of lipidated proteins, the vector pETLip3 (kindly provided by Intervet MSD, Boxmeer, The Netherlands) was used, which contains the signal sequence of the outer surface protein A (*ospA*) from *Borrelia burgdorferi* (Figures S1 and S2). The corresponding genes for *dacB* (*sp_0629* in TIGR4; nt 61–715, aa 21–238) and *pnrA* (*sp_0845* in TIGR4; nt 67–1050, aa 23–350) were amplified by polymerase chain reaction (PCR) without the signal sequences from genomic DNA of the pneumococcal strain TIGR4 using specific oligonucleotide pairs listed in Table 1.

The PCR products were inserted into the pETLip3 vector using the specific restriction sites *Nde*I/*Bam*HI incorporated in the forward and reverse primers, respectively. For purification of lipidated proteins, reverse primers contained a sequence encoding for a C-terminal His_6_-tag. The constructs for the recombinant expression of non-lipidated, His_6_-tagged DacB and PnrA were previously described [5,9]. Recombinant plasmids were transformed into *E. coli* ClearColi^®^ (Lucigen^®^, Middleton, WI, USA), which were cultured in LBmodi medium (1% Bacto-Trypton, 0.5% yeast extract, 1% NaCl, pH 7.5). Expression of His_6_-tagged proteins was induced with 1 mM IPTG. After lysis of *E. coli* ClearColi^®^ by sonication, lysates were either incubated with 20 mM zwitterionic detergent CHAPS [3-((3-cholamidopropyl)dimethylammonio)-1-propanesulfonate, Carl Roth, Karlsruhe, Germany] for solubilization of lipidated proteins or directly centrifuged followed by affinity chromatography of supernatants using HisTrap™ Ni-NTA columns in the “ÄKTA purifier liquid chromatography system” (GE Healthcare GmbH, Freiburg im Breisgau, Germany). Purified proteins were dialyzed against phosphate-buffered saline (PBS). Following SDS-PAGE, purity of the proteins was analyzed using silver staining and immunoblotting. Immunoblots were performed with antigen-specific mouse antisera (α-DacB, α-PnrA) and IRDye^®^ 800CW goat anti-mouse IgG followed by detection using the Odyssey^®^ CLx Imaging System (LI-COR^®^, Bad Homburg, Germany).

### 2.2. Top-Down Analysis of Recombinant Proteins with LC–MS

Recombinant lipidated proteins were verified by liquid chromatography–mass spectrometry (LC–MS). In a top-down analysis, protein samples were directly loaded onto the online desalting system AdvanceBio Desalting-RP (Agilent, Santa Clara, CA, USA). The LC–MS system consisted of an HPLC 1100 (Agilent, Santa Clara, CA, USA) coupled to an electrospray ionization (ESI) mass spectrometer, Q-Tof Ultima (Waters, Milford, MA, USA). The proteins were injected into the high-performance liquid chromatography (HPLC) with aqueous 0.1% formic acid. Separation and elution of the lipidated proteins were enabled by gradually increasing the hydrophobicity with 0.1% formic acid in acetonitrile. The mass spectra were externally calibrated with bovine ubiquitin (Sigma-Aldrich, Munich, Germany). Molecular weights (MWs) of proteins were determined using ESIprot online for MS spectra deconvolution [24].

### 2.3. Surface Localization of Heterologously Expressed Proteins on E. coli ClearColi

To detect the heterologously expressed lipidated proteins on the surface of *E. coli* ClearColi^®^, bacteria were sedimented and resuspended in PBS. Bacteria (2 × 10^8^) were incubated with antigen-specific mouse antisera (α-DacB, α-PnrA) for 45 min at 4 °C. After washing, bacteria were incubated for 45 min at 4 °C with Alexa Fluor 488-coupled goat anti-mouse IgG. Bacteria were fixed with 1% paraformaldehyde overnight at 4 °C. Flow cytometry was conducted with FACSCalibur™ (BD Biosciences, Heidelberg, Germany). The data were acquired by CellQuestPro Software 6.0 (BD Biosciences, Heidelberg, Germany) and analyzed with Flowing Software 2.5.1 (Perttu Terho, Cell Imaging Core, Turku Centre for Biotechnology, Turku, Finland).

### 2.4. Mouse Model for Immunization and Pneumococcal Colonization

Animal experiments were conducted according to the Dutch law and with the approval of the Radboud University Medical Center Committee for Animal Ethics (Ethic approval code AVD103002017904). Six- to eight-week-old female inbred C57BL/6 mice (Charles River Laboratories, Sulzfeld, Germany) were immunized intranasally (9 groups, *n* = 8) or subcutaneously (9 groups, *n* = 8) three times in a two-week interval on days 0, 14, and 28. Vaccine formulations contained 5 µg lipidated DacB (Lip-DacB), non-lipidated DacB (DacB), lipidated PnrA (Lip-PnrA), or non-lipidated PnrA (PnrA) in a total volume of 10 µL or 50 µL for intranasal or subcutaneous immunizations, respectively. While half of the groups per immunization route received vaccine formulations without adjuvant, in four of the groups, adjuvant was added. Either 4 µg Cholera toxin subunit B (CTB, Sigma-Aldrich Chemie GmbH, Taufkirchen, Germany) or 25 µL Imject™ Alum (ThermoFisher Scientific, Bonn, Germany) was used for intranasal or subcutaneous vaccinations, respectively. One group per immunization route (*n* = 8/group) received PBS as negative control. Tail vein blood samples were taken before each immunization step on days-3, 11, and 25 (*n* = 4/group), and two weeks after the last immunization on day 42 (*n* = 8/group) for antibody measurements. On day 49, three weeks after the final immunization, mice were intranasally infected under inhalation anesthesia with 10^6^ colony forming units (CFU) of *S. pneumoniae* PBCN0231 (serotype 4) in 5 µL. On day 52, three days post-infection, mice were euthanized, and blood, nasal tissue (*n* = 8/group), and spleens (*n* = 4/group) were harvested. The nasal tissue was transferred in 1 mL PBS and homogenized using the disperser T 10 basic ULTRA-TURRAX^®^ in combination with the S 10 N–5 G Dispersing tool (IKA^®^-Werke GmbH & CO. KG, Staufen im Breisgau, Germany). Nasal tissue homogenates were 10-fold serially diluted in PBS and plated on blood agar plates to determine the bacterial recovery (CFU/mL nasal tissue homogenate). Remaining nasal tissue homogenates were snap frozen in liquid nitrogen and stored at −80 °C until measurement of local cytokine levels. Isolated spleens were directly transferred to culture medium (RPMI 1640 Glutamax + 10% FCS + 1% Pen/Strep) and kept on ice until further processing for in vitro stimulation.

### 2.5. In Vitro Cell Stimulations

HEK-Blue-hTLR2-TLR1, HEK-Blue-hTLR2-TLR6, and HEK-Blue-hTLR2-KO-TLR1/6 cells carrying a SEAP reporter construct (InvivoGen, Toulouse, France) were grown in Gibco™ DMEM Glutamax (Fisher Scientific, Landsmeer, The Netherlands) supplemented with 10% FCS, 2 mM L-glutamine, 100 µg/mL Normocin in the presence of HEK-BLUE selective antibiotic according to the manufacturer’s recommendations. When the cells reached 50–80% confluency, they were detached using 0.05% Trypsin-EDTA (Lonza, Basel, Switzerland), washed once with PBS, resuspended in HEK-Blue detection medium (InvivoGen, Toulouse, France), and used directly. For the assay, 5 × 10^5^ cells were stimulated with serial dilutions of purified DacB, Lip-DacB, PnrA, and Lip-PnrA (1 µg/mL–1 ng/mL) in a final volume of 200 µL. Pam3CSK4 (EMC Microcollections, Tuebingen, Germany) and Pam2CSK4 (InvivoGen) were included as positive controls for HEK-Blue-hTLR2-TLR1 and HEK-Blue-hTLR2-TLR6 cells, respectively. After 15 h of stimulation at 37 °C, 5% CO_2_, the OD at 620 nm was measured.

For in vitro splenocyte stimulations, single cell suspensions of spleens (*n* = 4/group) were prepared in culture medium using a Falcon^®^ 70 µm cell strainer (Fisher Scientific, Schwerte, Germany) followed by incubation with red blood cell lysis buffer (Invitrogen, Karlsruhe, Germany) for 5 min. Splenocytes (5 × 10^5^ in 100 µL) were stimulated for 72 h at 37 °C and 5% CO_2_ with an equal volume of Lip-DacB or Lip-PnrA (1 µg/mL), matching the immunization background. Lipidated proteins were used for stimulation as these are the variants that are encountered during a natural infection. Cells of PBS-treated animals were stimulated either with lipidated DacB or PnrA as a negative control. Supernatants of stimulated splenocytes were collected and stored at −20 °C until measurement of systemic cytokine levels.

### 2.6. Measurement of Cytokine Levels

Cytokine production in undiluted mouse nasal tissue homogenates (*n* = 8/group) or supernatants of stimulated splenocytes (*n* = 4/group) were determined with LEGENDplex™ Mouse Th Cytokine Panel (13-plex, BioLegend, London, UK). Except for reducing the used volumes described in the manual by half, the assay was performed according to manufacturer’s instructions. Flow cytometry was conducted with a BD LSR II (BD Biosciences, Heidelberg, Germany). The data were acquired by BD FACSDiva™ (BD Biosciences) and analyzed with LEGENDplex™ Data Analysis Software (BioLegend, London, UK).

### 2.7. Detection of Antibody Responses by Enzyme-Linked Immunosorbent Assay (ELISA)

Non-lipidated proteins were immobilized in equimolar amounts (3 pmol) on Maxisorp plates (Laborhaus Scheller, Euerbach, Germany) overnight at 4 °C. For standard curves, wells were plated with increasing concentrations of mouse IgA, IgG, IgG1, IgG2b, IgG2c, or IgG3 isotype control. Free binding sites were blocked with 1% BSA. Plates were incubated for 1 h at 37 °C with 50 µL diluted nasal tissue or serum sample followed by incubation for 1 h with 50 µL diluted secondary antibody. Secondary antibodies used were peroxidase-coupled goat anti-mouse IgA, IgG, IgG2b, IgG2c, and IgG3 or rabbit anti-mouse IgG1. Antibody binding was detected with *o*-phenylenediamine dihydrochloride (0.7 mg/mL in water) supplemented with 0.03% hydrogen peroxide. The color reaction was stopped by 2 M sulphuric acid followed by measurement of the absorbance at 492 nm with the FLUOstar Omega microplate reader (BMG Labtech, Ortenberg, Germany).

### 2.8. Statistical Analyses

All statistical analyses were performed using GraphPad Prism version 5.0 (GraphPad Software, San Diego, CA, USA). One-way ANOVA Kruskal–Wallis test with Dunn’s post-test or one-way ANOVA with Bonferroni’s post-test were used to compare multiple groups. The Mann–Whitney *U*-test on original data or one-sided *t*-test on log-transformed data was used to compare two groups. Spearman correlation coefficients were calculated with control animals excluded to avoid bias of the strength of correlation.

Multivariate redundancy analysis (RDA) was done using Canoco 5.11 with default settings of the analysis type ‘Constrained’ [25]. RDA calculates *p*-values by randomly permuting the sample status and thereafter counts the number of times a permuted set of samples has a better separation compared to the non-permuted RDA model. Differences with a *p*-value < 0.05 were considered statistically significant.

## 3. Results

### 3.1. Production of Recombinant Lipidated Proteins

**Figure 1:**
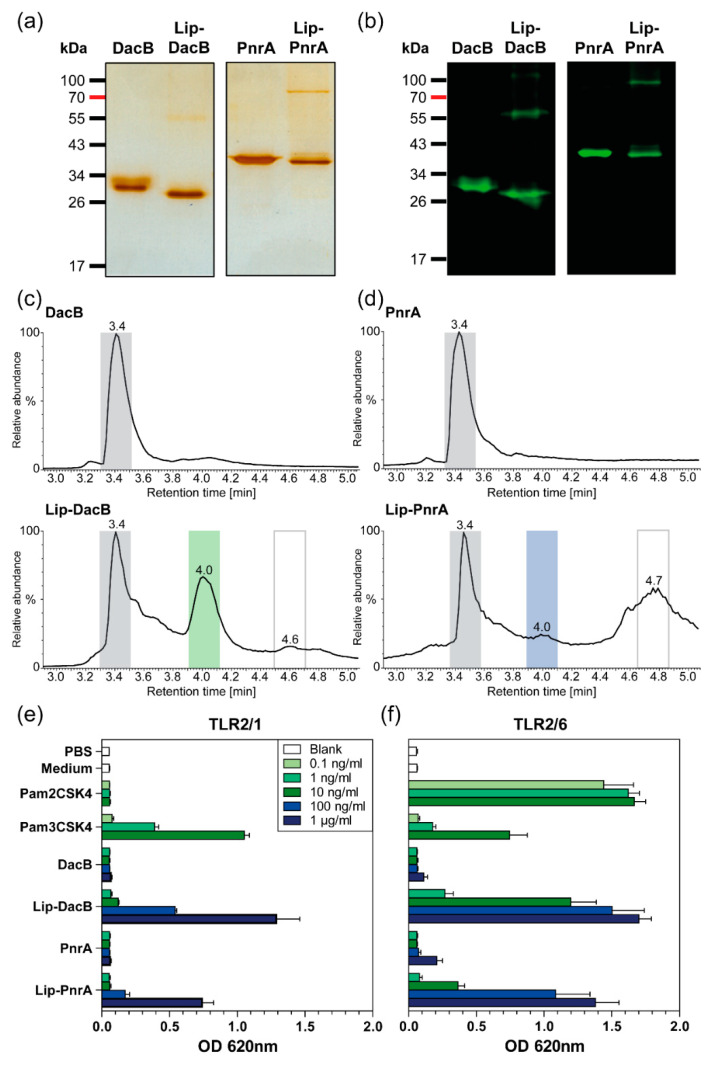
Characterization of lipidated and non-lipidated DacB and PnrA. Purified recombinant lipidated and non-lipidated DacB and PnrA (each 1 µg) were separated by SDS-PAGE (12%) and detected either by silver staining for validation of protein purification (**a**) or by immunoblots using lipoprotein-specific antibodies (**b**). Total ion current chromatograms from HPLC of lipidated and non-lipidated DacB (**c**) and PnrA (**d**). Separation and elution of the lipidated proteins was enabled by gradually increasing the hydrophobicity with 0.1% formic acid in acetonitrile. The peaks at retention time (RT) 3.4 min and RT 4.0 min reflect non-lipidated and lipidated proteins, respectively. The peak at RT 4.6/4.7 min represents phospholipids from the cell membrane. HEK-Blue-hTLR2-TLR1 (**e**) and HEK-Blue-hTLR2-TLR6 (**f**) cells were stimulated with increasing doses of Pam2CSK4, Pam3CSK4, lipidated, and non-lipidated proteins DacB or PnrA. Following stimulation in HEK-Blue detection medium over 15 h, the OD at 620 nm was measured. Mean results of three independent experiments are shown with error bars corresponding to SD. Equally stimulated HEK-Blue-hTLR2-KO-TLR1/6 cells showed signals corresponding to the negative controls (Figure S4).

Recombinant lipidated proteins Lip-DacB and Lip-PnrA were produced in *E. coli* ClearColi^®^ by the insertion of encoding genes for *dacB* and *pnrA* in the expression vector pETLip3. The genes were cloned downstream of the plasmid-encoded signal sequence of the gene coding for the outer surface protein A (*ospA*) from Gram-negative *Borrelia burgdorferi* that enabled N-terminal lipidation of proteins [26]. Heterologously expressed lipidated proteins were solubilized by treatment with the zwitterionic detergent CHAPS and purified using affinity chromatography based on a C-terminal His_6_-tag. Expression and purification of non-lipidated proteins were performed as described previously [5,9]. Purity of recombinant proteins was verified by SDS-PAGE (Figure 1a). Strikingly, in addition to the protein bands corresponding to the molecular weights (MWs) of the lipidated proteins, SDS-PAGE revealed protein species with double MWs. Immunoblots with antigen-specific antibodies confirmed that the lipidated proteins form dimers, possibly due to interactions of the lipid moieties via van der Waals forces (Figure 1b).

The OspA of *Borrelia burgdorferi* is targeted to the outer surface by default [27]. Accordingly, heterologous lipidation of DacB and PnrA in *E. coli* should similarly lead to their cell-surface exposure. Therefore, *E. coli* ClearColi^®^ strains expressing lipidated or non-lipidated DacB or PnrA were analyzed by flow cytometry using antigen-specific antibodies (Figure S3). Targeting of lipidated proteins to the surface was confirmed, thereby approving successful lipidation. However, fluorescence signals for ClearColi^®^ expressing Lip-DacB were higher compared to Lip-PnrA, suggesting that lipidation was more efficient for Lip-DacB.

### 3.2. Chemical Characterization and TLR2 Targeting of Recombinant Lipidated Proteins

Gas chromatography–mass spectrometry (GC–MS) analysis of purified heterologously expressed lipidated OspA revealed saturated fatty acids ranging from C14–C18 as well as C16 and C18 unsaturated fatty acids, with palmitate (C16:0) being the major component [28]. Therefore, the main form of N-terminal lipidation represents a *S*-[2,3-bis(palmitoyloxy)-(2*R,S*)-propyl]-*N*-palmitoylcysteine modification, which is consistent with the model established by Hantke and Braun [29]. To further confirm lipidation of DacB and PnrA, lipidated proteins were analyzed by LC–MS (Figure 1c,d; Figures S5 and S6). The results of the analyses are summarized in Table 2.

According to the chromatograms from HPLC, preparations of non-lipidated proteins showed one peak at a retention time (RT) of 3.4 min corresponding to non-lipidated DacB and PnrA (Figure 1c,d). The elution profiles of lipidated DacB and PnrA were composed of three major components. The lipidated protein fractions could be detected at RT 4.0 min with masses corresponding to tri-palmitoylated proteins. The protein species at RT 3.4 min potentially represented a post-translationally unmodified protein still carrying the OspA signal sequence (Figures S5 and S6). With respect to the yield, considerable amounts of lipidated protein could be achieved for Lip-DacB (Figure 1c), which is indicated by a relatively high ratio of 0.67:1 of the lipidated (RT:4.0 min) to non-lipidated protein fraction (RT:3.4 min). The amount of Lip-PnrA was significantly lower with a ratio of 0.26:1 (Figure 1d).

Lipidated and non-lipidated proteins DacB and PnrA were used to stimulate HEK293 reporter cells that are stably transfected with one of the heterodimers TLR2/1 or TLR2/6, respectively (Figure 1e,f; Figure S4). In these assays, lipidated proteins activated both TLR2/1 and TLR2/6 in a dose-dependent manner confirming engagement of TLR2 by the lipidated proteins. Activation by Lip-PnrA, however, was consistently lower compared to Lip-DacB. Non-lipidated proteins elicited responses in HEK TLR2 cells comparable to the negative controls.

### 3.3. Protein Lipidation Enhances the Systemic Antibody Response in an Antigen-, Administration Route-, and Adjuvant-Dependent Manner

**Figure 2:**
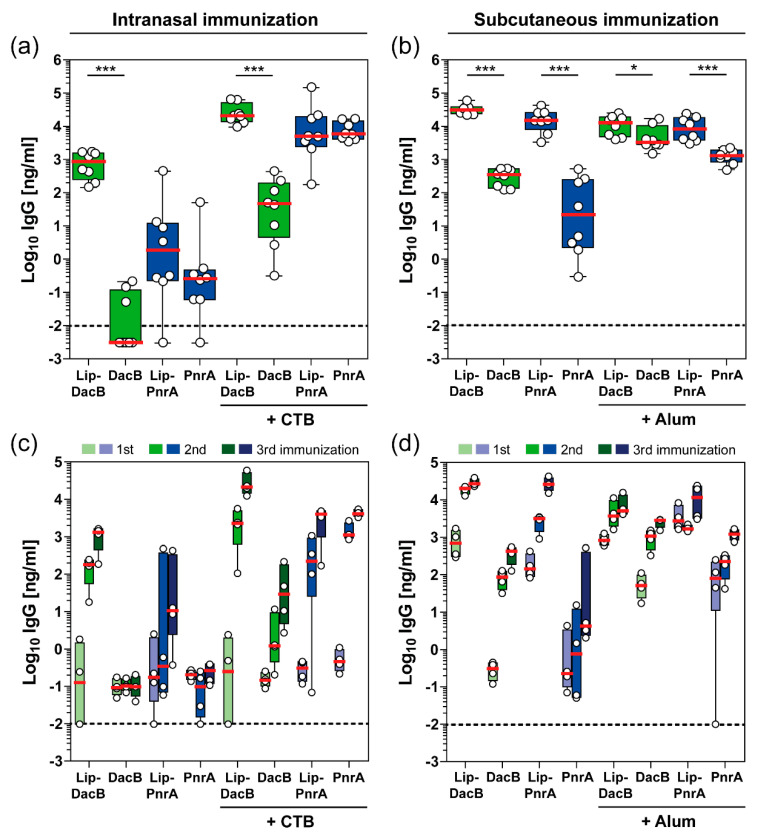
Impact of antigen lipidation on the systemic antibody response in mice. C57BL/6 mice (*n* = 8/group) received three intranasal (**a**,**c**) or subcutaneous (**b**,**d**) vaccinations with 5 µg lipidated or non-lipidated DacB or PnrA with or without additional adjuvant. Before each immunization and two weeks after the third immunization, sera were collected and monitored for their antigen-specific IgG concentrations using ELISA. Antigen-specific IgG levels (Log_10_ ng/mL) in post-immune sera of all mice two weeks after the final immunization (**a**,**b**) and kinetics of IgG levels (Log_10_ ng/mL) following each vaccination of four randomly selected mice per group (**c**,**d**) are shown. Box plots represent group median (horizontal red line), first and third quartiles (box), and the range of data (whiskers). Symbols represent individual mice. The dashed line indicates the lower limit of detection. Statistical significance was determined using a Mann–Whitney U test. *, *p* < 0.05; ***, *p* < 0.001.

The lipidated and non-lipidated proteins were used for intranasal or subcutaneous vaccinations of C57BL/6 mice to study the impact of lipidation on the induced humoral immune responses. In addition, mice were included that received an adjuvanted (CTB or Alum) formulation to study whether this, together with lipidated antigens, leads to an enhanced humoral immune response or whether lipidation itself is sufficient to potentiate antigen-specific antibody production (Figure 2).

Lipidation of DacB substantially impacted the magnitude of the induced systemic IgG responses following intranasal immunization, irrespective of the use of CTB (Figure 2a). Non-lipidated DacB elicited only marginal IgG responses when administered intranasally with or without adjuvant. Intranasal vaccination with Lip-DacB increased the final IgG concentrations by five (without CTB) or three orders (with CTB) of magnitude, whereas for PnrA, the effect of lipidation, also in the presence of CTB, was minor. Notably, following subcutaneous immunization, protein lipidation resulted in a statistically significant increase in antigen-specific IgG responses in all groups (Figure 2b). IgG responses to both lipidated proteins were even higher than to alum-adjuvanted non-lipidated proteins. IgG levels measured in pre-immune sera were negligible and close to the detection limit (Figure S7). Interestingly, lipidation stimulated antibody production but did not create new epitopes for antibody binding, as indicated by similar IgG levels detected in the ELISA when using lipidated or non-lipidated protein for immobilization (Figure S8). Therefore, lipidation enhances the immunogenicity of the coupled antigens but the lipid moiety itself is not immunogenic.

The effect of lipidation on the antibody kinetics over the course of each immunization was analyzed for four mice per group (Figure 2c,d). Interestingly, the influence of lipidation on antibody kinetics was primarily dependent on the immunization route. While IgG levels in the intranasal immunization were still comparable between lipidated and non-lipidated proteins after priming, the following booster immunizations significantly increased IgG levels in the presence of lipidation (Figure 2c). In contrast, following subcutaneous immunization, lipidation had a major impact on IgG levels after the priming immunization (Figure 2d). Strikingly, IgG levels induced by Alum-adjuvanted lipidated PnrA already exceeded the final IgG concentrations induced by the Alum-adjuvanted non-lipidated PnrA after the first immunization. In conclusion, lipidation had a substantial impact on the magnitude and kinetics of the induced humoral immune response, which was dependent on the antigen, vaccination route, as well as the adjuvant used.

### 3.4. Protein Lipidation Induces IgG Subclass Responses Related to Th1-Type Immunity

**Figure 3:**
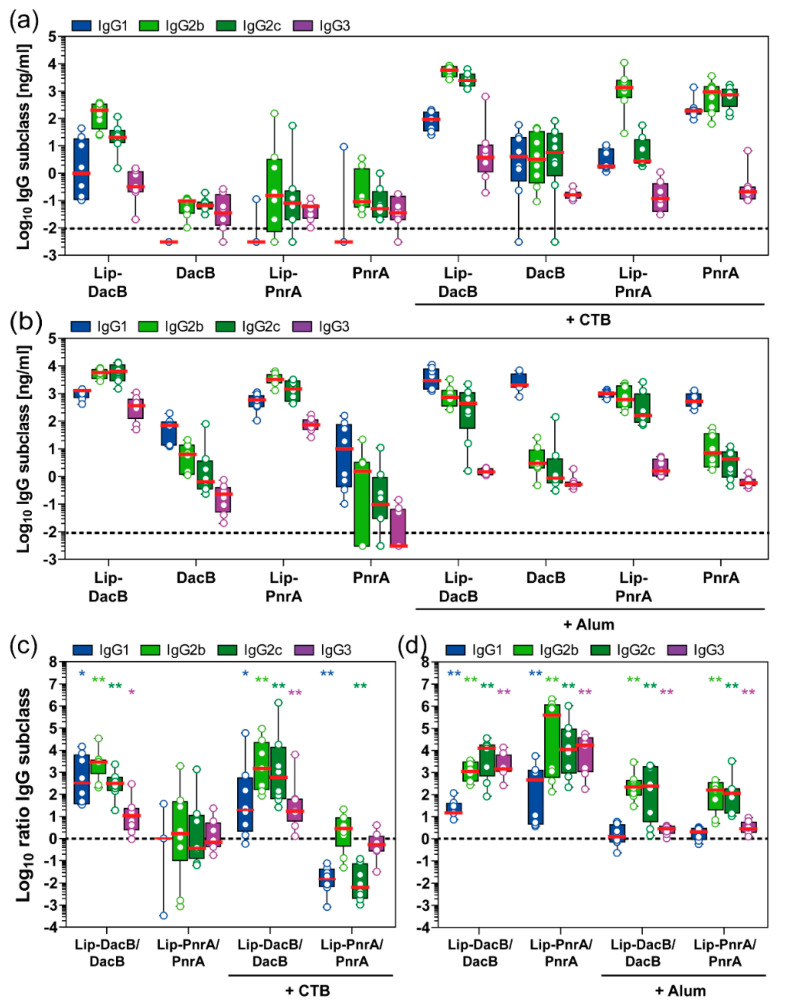
Impact of antigen lipidation on the systemic IgG subclass response in mice. Following intranasal (**a**,**c**) or subcutaneous (**b**,**d**) immunization of mice (*n* = 8/group), post-immune antisera were monitored for their antigen-specific IgG1, IgG2b, IgG2c, and IgG3 concentrations (Log_10_ ng/mL) using ELISA. IgG subclass ratios (Log_10_) of lipidated to non-lipidated DacB or PnrA are shown to visualize the impact of lipidation on the type of the induced antibody response (**c**,**d**). Box plots represent group median (horizontal red line), first and third quartiles (box), and the range of data (whiskers). Symbols represent individual mice. The dashed line either indicates the lower limit of detection (**a**,**b**) or the ratio at equilibrium (**c**,**d**). Wilcoxon signed rank test was used to check whether the group medians (**c**,**d**) were significantly different from equilibrium (ratio 1:1). *, *p* < 0.05; **, *p* < 0.01.

To elucidate the lipidation-induced T helper (Th) cell responses, IgG subclasses (IgG1, IgG2b, IgG2c, IgG3) were measured in post-immune mouse sera (Figure 3a,b), while ratios of IgG subclass concentrations of lipidated to non-lipidated (Lip:NonLip) DacB or PnrA were calculated to focus on the lipidation-induced effects (Figure 3c,d).

Overall, intranasal immunization with non-lipidated proteins and CTB induced a balanced IgG2/IgG1 response with marginal IgG3 levels (Figure 3a). However, lipidation of DacB significantly increased Lip:NonLip ratios of IgG1, IgG2b, IgG2c, and IgG3 by 20-, 1400-, 500-fold, and 16-fold, respectively, indicating that lipidation not only enhanced antibody responses but also modulated the induced type of IgG subclass response towards IgG2 (Figure 3c). Interestingly, lipidation of PnrA resulted in an opposite effect following intranasal immunization with CTB. IgG subclass responses were dominated by IgG2b (Figure 3a). However, compared to non-lipidated PnrA, significantly lower levels of IgG1 and IgG2c were detected, with Lip:NonLip ratios of IgG1 and IgG2c reduced by 60- and 150-fold, respectively (Figure 3c).

Subcutaneous immunization with non-lipidated proteins in combination with Alum induced mainly antibodies of the IgG1 subclass, while levels of IgG2b, IgG2c, and IgG3 were significantly lower (Figure 3b). However, when combined with Alum, lipidation of both proteins tested significantly increased Lip:NonLip ratios of IgG2b, IgG2c, and IgG3 by 190-, 180-fold, and 3-fold, respectively, while ratios of IgG1 remained unaltered (Figure 3d). Lipidated proteins without Alum induced a similar pattern with increased levels of IgG2b and IgG2c and additionally an enhanced production of IgG1 compared to the non-lipidated proteins (Figure 3b,d). In addition, IgG3 concentrations for Lip-DacB and Lip-PnrA were significantly elevated, reaching 250 and 46 times higher levels, respectively, compared to the corresponding Alum-adjuvanted groups (Figure 3b).

In conclusion, dependent on the antigen, vaccination route, and adjuvant used, lipidation skewed the IgG subclass responses towards IgG2 and partially IgG3, thus inducing IgG subclass responses related to a Th1-type immunity.

### 3.5. Intranasal Immunization with Lipidated Proteins Enhances the Mucosal Antibody Response in the Nasal Tissue

**Figure 4:**
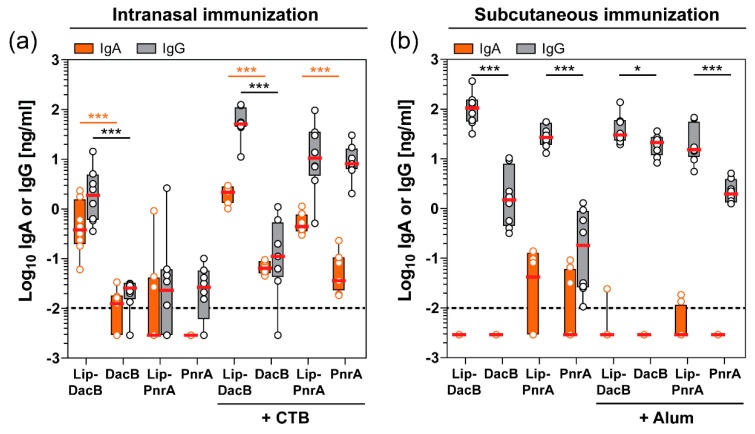
Impact of antigen lipidation on the local antibody response in the nasal tissue of mice. Intranasally (**a**) or subcutaneously (**b**) immunized C57BL/6 mice (*n* = 8/group) were intranasally infected with *S. pneumoniae* for three days followed by monitoring of nasal tissue homogenates for their antigen-specific IgA and IgG concentrations (Log_10_ ng/mL) using ELISA. Box plots represent group median (horizontal red line), first and third quartiles (box), and the range of data (whiskers). Symbols represent individual mice. The dashed line indicates the lower limit of detection. Statistical significance was determined using a Mann–Whitney U test. *, *p* < 0.05; ***, *p* < 0.001.

Since *S. pneumoniae* colonizes the mucosal surface of the upper respiratory tract, it is important to determine antigen-specific antibodies available at this site of infection as they might play a role in the containment of this pathogen [21,22]. Therefore, the nasal tissue of immunized mice that were subsequently colonized with pneumococci for three days were assessed for mucosal antigen-specific IgA and IgG levels (Figure 4).

Although substantially lower IgG concentrations were measured in the nasal tissue, mucosal levels showed a consistent pattern compared to the systemic levels. Significant mucosal IgA levels could only be detected following intranasal immunization, whereas subcutaneous immunization did not induce a detectable IgA response. Following intranasal immunization, lipidation significantly increased local IgA levels, with the exception of Lip-PnrA (Figure 4a). Interestingly, lipidation of PnrA enhanced the IgA response when formulated with CTB, while local IgG levels were not affected. These data show that protein lipidation is able to potentiate the systemic as well as the mucosal humoral immune response.

### 3.6. Local and Systemic Cytokine Responses Are Not Significantly Driven by Protein Lipidation

Local and systemic cytokine profiles induced by immunization and subsequent pneumococcal colonization were analyzed to elucidate whether immunization with lipidated proteins leads to differences in cellular immunity upon encountering *S. pneumoniae* compared to non-lipidated proteins. Therefore, cytokine levels were determined in the nasal tissue homogenates (of intranasally immunized mice) and in supernatants of in vitro restimulated splenocytes (for both immunization routes) from immunized and pneumococcal colonized mice, respectively.

Nasal tissue homogenates of intranasally immunized and pneumococcal colonized mice revealed a tendency of any vaccine formulation to induce increased levels of pro-inflammatory cytokines Interferon-γ (IFN-γ) and tumor necrosis factor alpha (TNF-α) compared to PBS-treated mice (Figure S9). In the CTB-adjuvanted groups, significantly increased levels of local IL-17A could be detected independent of lipidation.

**Figure 5:**
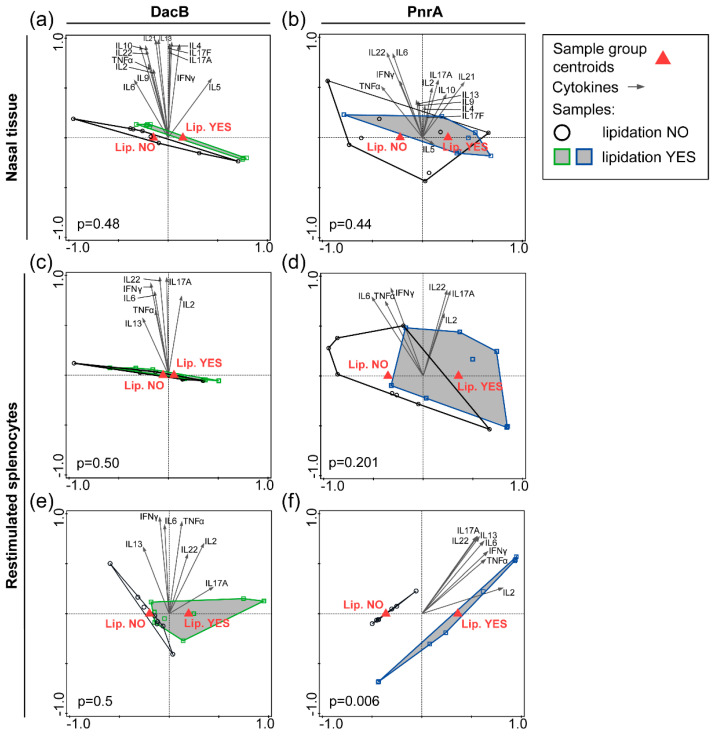
Impact of lipidation on the cytokine response in the nasal tissue or in in vitro restimulated splenocytes of immunized and pneumococcal colonized mice. Nasal tissue of C57BL/6 mice (*n* = 8/group) that were intranasally immunized with lipidated or non-lipidated DacB (**a**) or PnrA (**b**) followed by an intranasal infection with *S. pneumoniae* three days prior to collection was analyzed. Cytokine levels in tissue homogenate were used for RDA to determine local lipidation-specific cytokine signatures for both proteins. Splenocytes were obtained from C57BL/6 mice (*n* = 4/group) that were intranasally (**c**,**d**) or subcutaneously (**e**,**f**) immunized followed by the pneumococcal infection. Cells were restimulated in vitro with lipidated proteins, matching the vaccination background, for 72 h. The cytokine levels were assessed and used for RDA irrespective of additional adjuvantation to evaluate systemic lipidation-specific cytokine signatures for DacB (**c**,**e**) and PnrA (**d**,**f**). Lipidation YES refers to mice vaccinated with lipidated proteins; and Lipidation NO refers to mice vaccinated with non-lipidated proteins. Arrows indicate individual cytokines, red triangles the sample group centroids, and squares/circles represent individual mice. *p*-values are shown; *p* < 0.05 is considered significant.

Subsequently, nasal cytokine levels were used for supervised RDA to explore whether certain cytokines correlated with either protein lipidation or adjuvantation (Figure 5a,b; Figure S10a,b). Significant differences in cytokine profiles were observed between groups with and without CTB adjuvant, with nasal IL-17A being the main driver for both proteins, i.e., DacB and PnrA (Figure S10a,b). However, the RDA revealed no lipidation-associated cytokine profile when comparing the lipidated and non-lipidated proteins (Figure 5a,b).

For the systemic cytokine profiles induced by immunization and subsequent pneumococcal colonization, splenocytes were stimulated in vitro with either lipidated DacB or PnrA, matching the vaccination background of the animal (Figure S11). Similar to the nasal cytokine profiles, elevated systemic IL-17A levels were detected following intranasal immunization exclusively in the CTB-adjuvanted groups independent of lipidation (Figure S11a). Following subcutaneous immunization, Lip-PnrA in combination with Alum showed significantly increased levels of IFN-γ, IL-6, IL-2, IL-17A, IL-22, and IL-13 compared to PBS-treated mice (Figure S11b).

As with nasal cytokine levels, systemic cytokine levels were used for RDA to explore whether certain cytokines correlated with either protein lipidation or adjuvantation (Figure 5c–f, Figure S10c–f). Thus, RDAs were performed per antigen using either the data from mice receiving lipidated antigens compared to the non-lipidated variants, irrespective of the presence of an adjuvant (Figure 5c–f), or comparing the data of adjuvanted and non-adjuvanted mice, independent of protein lipidation (Figure S10c–f). RDAs showed no significant differences in cytokine profiles between mice intranasally vaccinated with lipidated or non-lipidated proteins (Figure 5c,d), whereas co-administration with CTB did (Figure S10c,d), which is in line with the nasal cytokine signature. For subcutaneous immunized mice, RDA showed that both PnrA lipidation and co-administration with Alum significantly changed the cytokine profile compared to no lipidation and no Alum, respectively, which was not the case for DacB (Figure 5e,f; Figure S10e,f). This suggests that the vaccination background, including vaccination route, adjuvant, and antigen, steers the splenocyte cytokine response. However, the data mainly indicate that local and systemic cytokine responses are not strongly affected by protein lipidation.

### 3.7. Immunization with Lipidated Antigens Moderately Reduces the Nasal Bacterial Load Compared to Non-Lipidated Variants

**Figure 6:**
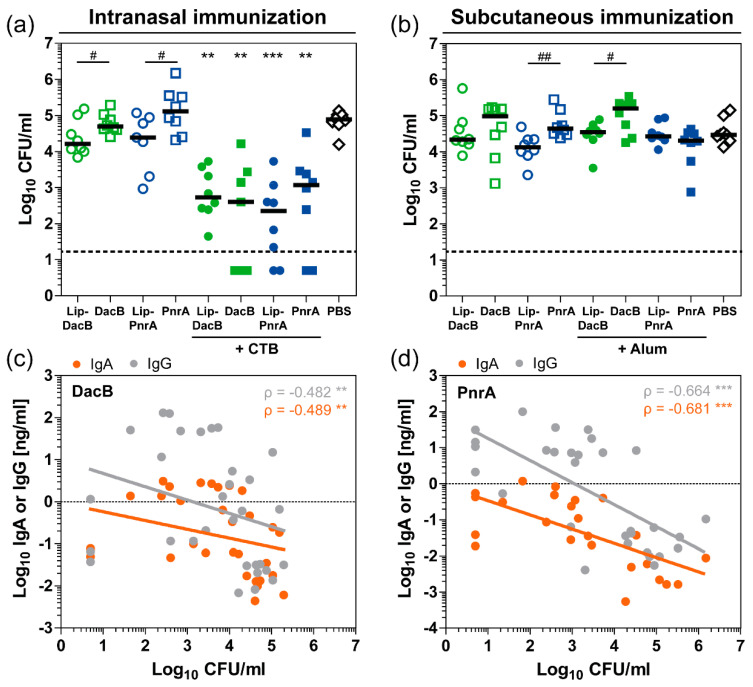
Protein lipidation as well as adjuvantation with CTB reduce the nasal bacterial load, which correlates inversely with nasal antibody levels. The bacterial load (Log_10_ CFU/mL) in nasal tissue of C57BL/6 mice three days post intranasal infection with 10^6^ CFU *S. pneumoniae* was determined following three intranasal (**a**) or subcutaneous (**b**) immunizations with 5 µg of lipidated or non-lipidated DacB or PnrA with or without additional adjuvant. Symbols represent individual mice, solid lines the group median, and the dashed line indicates the lower limit of detection. Data were statistically analyzed either by a Kruskal–Wallis test accompanied by Dunn’s multiple comparison post-test, with all conditions compared to PBS-treated mice to compare multiple groups (**, *p* < 0.01; ***, *p* < 0.001) or by one-sided *t*-test on log-transformed data to compare two groups (^#^, *p* < 0.05; ^##^, *p* < 0.01). Paired analysis of CFU counts and nasopharyngeal IgA and IgG levels of intranasally DacB- (**c**) or PnrA-immunized (**d**) mice with data of vaccinations with lipidated or non-lipidated DacB or PnrA combined, respectively. Symbols represent individual mice (*n* = 8/group). Analysis was performed on log-transformed data. Spearman’s coefficients (ρ) and *p*-values are shown (**, *p* < 0.01; ***, *p* < 0.001). The coefficients of determination (R²) of the linear regression for DacB are 0.094 (IgA) and 0.090 (IgG) and for PnrA, 0.439 (IgA), 0.461 (IgG).

Next, we investigated whether the immunological differences induced by vaccination with either lipidated or non-lipidated proteins have an impact on the level of protection against pneumococcal colonization. Therefore, three weeks after the last immunization, mice were intranasally infected with *S. pneumoniae*. Three days post-infection, mice were euthanized and viable pneumococci were recovered from the nasal tissue (Figure 6a,b). Compared to PBS-treated mice, intranasal vaccination with CTB-adjuvanted proteins induced a significant reduction of the bacterial load in the nasal cavity (Figure 6a). Vaccination with Lip-PnrA + CTB revealed the strongest effect, causing a 350-fold reduction in nasal bacterial load. The impact of lipidation on protection was evaluated independently of the antigen by comparing lipidated and non-lipidated proteins. Lipidated DacB and PnrA significantly reduced the bacterial load by three and five times, respectively, compared to the non-lipidated variant when no adjuvant was used (one-sided *t*-test; p[DacB:Lip-DacB] = 0.0306 and p[PnrA:Lip-PnrA] = 0.0167). Compared to PBS-treated mice, subcutaneously immunized mice were not significantly protected against colonization with any of the vaccine formulations (Figure 6b). However, protein lipidation tended to reduce the pneumococcal load in the nasopharynx, which reached statistical significance for PnrA versus Lip-PnrA with a 3-fold and Alum-adjuvanted DacB versus Lip-DacB with a 4.5-fold reduction (one-sided *t*-test; *p* = 0.0024 and *p* = 0.0153, respectively).

Increased mucosal antibody responses were detected in the nasal tissue that were significantly impacted by lipidation (Figure 4). Following intranasal immunization, elevated levels of nasopharyngeal IgA and IgG correlated inversely with the nasal bacterial load (Figure 6c,d), which was not the case for the subcutaneous immunization (Figure S12). However, serum-derived IgG may have had a significant impact on colonization, since it can reach the surface by paracellular diffusion through the epithelium [30]. Based on correlations of systemic IgG subclass levels and nasal bacterial loads, subclasses IgG2b and IgG2c in particular might have contributed to the reduction in the bacterial loads (Figure S13). Of note, correlations of antibody levels reached higher significance for PnrA-immunized compared to DacB-immunized mice. In addition, nasopharyngeal IL-17A levels that were increased in the CTB-adjuvanted groups following intranasal immunization inversely correlated with protection (Figure S14b,c).

Therefore, our data indicate an important role of a combination of IL-17A and antibodies in protecting against pneumococcal colonization, the latter being significantly impacted by protein lipidation.

## 4. Discussion

Pneumococcal lipoproteins are of major interest for the development of a broadly protective protein-based vaccine. However, when produced recombinantly, these proteins lack their lipid moiety. In the present study, we investigated whether the immunogenicity and the protection induced by pneumococcal lipoproteins could be further improved by attaching a lipid anchor. To this end, lipidated and non-lipidated DacB and PnrA were produced and evaluated in a mouse immunization and colonization model.

Protein lipidation was characterized and validated by immunoblots, flow cytometry, a top-down LC–MS analysis, and a TLR2 stimulation assay. In LC–MS, for lipidated DacB, a molecular weight was identified that corresponds to an N-terminal tri-palmitoylation of the protein. However, slightly different molecular weights were found for lipidated PnrA. Although palmitic acid is generally the most predominant fatty acid, similar molecules within the cell envelope may serve as an acyl donor: palmitoleic acid, *cis*-vaccenic acid, 9,10-methylene-hexadecanoic acid, and stearic acid, potentially explaining the observed variability in lipidation [29]. In addition, the differences in the molecular weights determined by mass spectrometry may be a result of partial oxidations in the protein backbone [28,31]. In the TLR2 stimulation assay, both TLR2/1 and TLR2/6 heterodimers were stimulated by either of the two lipidated proteins. Although triacylated proteins are recognized by TLR2/1, there are studies indicating that activation through TLR2 can occur independent of TLR1 and TLR6 [32]. Lipidation of DacB was more efficient than that of PnrA, although in both cases a substantial amount of lipidated protein was produced. Interestingly, it is currently unknown how these differences in lipidation efficiency relate to the wild-type lipidation of these proteins in *S. pneumoniae*. Moreover, differences in lipidation mechanisms between Gram-positive and Gram-negative bacteria might also have influenced the lipidation efficiency of our heterologous lipidation method [33]. Although highly speculative, the observed differences in lipidation efficiency and/or moieties between DacB and PnrA could possibly be a consequence of differences in amino acid features following the lipidation sequence. Importantly, these differences between DacB and PnrA might have contributed to the protein-specific effects of lipidation presented in this study.

A major finding of the present study is the improved antibody kinetics and levels following immunization with lipidated proteins compared to non-lipidated proteins. This underpins that lipidated DacB and PnrA possess self-adjuvanting activity. The most prominent differences in serum IgG level between mice immunized with lipidated or non-lipidated proteins were observed after the first intranasal booster vaccination or the priming subcutaneous vaccination. Possible explanations for this difference between vaccination routes could be that (i) subcutaneous and intranasal vaccination require a distinct antigen dose for the induction of comparable antibody levels, (ii) B cells recognizing lipoproteins via TLR2 are activated and differentiate more efficiently following subcutaneous versus intranasal vaccination [34] and/or (iii) the sites of antigen deposition contain different densities and types of antigen presenting cells, which vary in TLR2 expression level and, when activated, might differentially stimulate B cell responses [35]. Interestingly, the enhanced antibody response was not a consequence of newly created epitopes by protein lipidation. Of note, next to the administration route, antibody induction was highly dependent on the adjuvant and antigen used, as has been described before [36,37]. However, antibody kinetics were only measured for a small number of mice per group (*n* = 4) and conclusions should therefore be interpreted with caution.

In addition to total IgG levels, protein lipidation influenced the antibody subclass distribution. When comparing antibody subclasses induced by vaccination with lipidated proteins to non-lipidated proteins, a major increase in IgG2b and IgG2c and a smaller increase in IgG3 were found, except for intranasally administered PnrA. Therefore, our data show that protein lipidation increases the IgG2/IgG1-ratio. This suggests that lipidation promotes skewing of the immune response towards a Th1 phenotype, which has been associated with a protective anti-pneumococcal immune response [38,39]. Moreover, murine IgG2 is found to be the most potent activator of effector responses, including complement fixation, and might therefore be favorable for protection against *S. pneumoniae* [40,41].

The TLR2-mediated immune-modulating activity of protein lipidation demonstrated here is in accordance with previously published studies. For instance, TLR2 signaling was shown to be important for enabling a Th1-related IgG response to pneumococcal infection in mice [16]. Similarly, lipidated Ag473 from serogroup B *Neisseria meningitidis* skewed T cell polarization towards a Th1 phenotype compared to non-lipidated Ag473 [42]. Besides TLR2, it is possible that Th1-biased immune responses of lipidated DacB and/or PnrA are due to engagement of TLR4 receptors, which may lead to profound differences in the initiation of downstream signaling pathways [43,44,45].

In contrast to the pronounced effect of lipidation on the humoral immune response, we found hardly any effect on the cytokine responses, as almost none of the RDAs were statistically significant. Co-administration of CTB in intranasal vaccine formulations increased the systemic and nasal IL-17A levels and, consistent with previous studies, the nasal IL-17A concentrations correlated inversely with the nasal pneumococcal load [15,46,47]. Interestingly, Th17 responses were found to be promoted by TLR2 activation in dendritic cells as well as CD4^+^ T cells, and Th17-mediated protection of a pneumococcal whole cell vaccine against carriage was dependent on TLR2 and surface-exposed lipoproteins [20,48,49]. In addition, in vitro stimulation of whole blood from mice immunized with lipidated proteins has been shown by others to induce elevated IL-17A levels compared to mice immunized with non-lipidated proteins [19]. However, we could not demonstrate an effect of protein lipidation on IL-17A levels. These discrepancies might be explained by differences in the experimental setup, including cell type, stimuli, and adjuvant used. Moreover, the cytokine signatures measured in this study should be interpreted with caution and subtle changes might have been missed, because the data were generated using a small number of animals (*n* = 4 for spleen cell stimulations). Furthermore, they are the result of a combined effect of the vaccine formulation used and the boosted immune response as a consequence of the pneumococcal infection. This combination of immunization and subsequent challenge also required the use of in vitro restimulated splenocytes as a measure of systemic inflammatory reactions instead of commonly used whole blood [19] due to a limited amount of blood samples.

Following pneumococcal challenge, solely mice that were intranasally vaccinated with CTB-adjuvanted proteins were significantly protected against pneumococcal colonization compared to PBS-treated mice, to a similar level as published previously for non-lipidated DacB and PnrA [9]. However, compared to the non-lipidated proteins, mice vaccinated with lipidated proteins showed the tendency to reduce the nasal bacterial load following both vaccination routes. This effect is comparable to that shown for lipidated versus non-lipidated MalX and GshT and suggests that the altered immune response due to lipidation is able to contribute to improved protection against colonization [19]. Despite the limited number of mice per group in the present study, we were able to reproduce the protective effect of the pneumococcal proteins DacB and PnrA, which was previously shown with a heterologous pneumococcal strain, underscoring the validity of our data [9].

In intranasally vaccinated mice, the nasal IgA and IgG levels, which were enhanced by protein lipidation, correlated inversely with the bacterial load. In addition, slightly reduced bacterial loads following subcutaneous immunization with lipidated proteins might likewise be attributed to a lipidation-induced increase in IgG levels. Although mucosal IgG levels of these subcutaneously vaccinated mice did not correlate with the reduced bacterial loads, serum-derived IgG translocated to the mucosal surface might have had an impact. It was found that especially the subclasses IgG2b and IgG2c correlated inversely with the reduced bacterial loads following both vaccination routes, indicating they may have contributed to the observed reduction. Of note, the unnaturally high challenge dose (10^6^ CFU) used for colonization might have partly hampered the measurement of the vaccine-induced immunity, leading to an underestimation of the role of antibody-mediated protection. Since antibodies have previously been shown to play a major role in protection, it is tempting to speculate that vaccination with lipidated antigens would result in a significantly better protection from invasive disease compared to vaccination with non-lipidated proteins [23,50].

## 5. Conclusions

In conclusion, we have shown that vaccination of mice with lipidated pneumococcal lipoproteins results in improved quantity and quality of antibodies compared to vaccination with non-lipidated proteins, but did not significantly influence the cellular immune response. Mice vaccinated with lipidated proteins showed a mild but encouraging reduction in nasal bacterial load compared to mice immunized with the non-lipidated variant. Therefore, lipidation of antigens represents a promising approach for the development of novel pneumococcal vaccine formulations, which has to be validated and extended in future studies using other pneumococcal antigens or combinations thereof.

## Figures and Tables

**Table 1:** Primer list.

Primer Name/Restriction Enzyme	Purpose	Sequence (5′-3′) *
DacB_pETLip_forw *Nde*I	Heterologous lipidation and expression of DacB (SP_0629)	5′-GCGCCATATGCAAGAAAAAACAAAAAATGAAGATGGAGAAACTAAGAC-3′
DacB_pETLip_rev *Bam*HI		5′-GCGGATCCTTA*ATGATGATGATGATGATG*ATCGACGTAGTCTCCGCC-3′
PnrA_pETLip_forw *Nde*I	Heterologous lipidation and expression of PnrA (SP_0845)	5′-GCGCCATATGGGTAACCGCTCTTCTCGTA-3′
PnrA_pETLip_rev *Bam*HI		5′-GCGCGGATCCTTA*ATGATGATGATGATGATG*TTTTTCAGGAACTTTTACGC-3′

* Restriction sites used for cloning are underlined. His_6_-tag in reverse primer is in italics.

**Table 2:** Theoretical and measured molecular weights of lipidated and non-lipidated proteins by LC–MS.

Protein	Theoretical Mass * [Da]	Retention Time [min]	Measured Mass [Da]	Mean Mass Difference ** [Da]
Non-lipidated proteins				
DacB	26,614	3.4	26,615.1 ± 2	+1.1
PnrA	36,890	3.4	36,895 ± 1.2 (major)	+5
			36,937 ± 1	+47
Lipidated proteins				
Lip-DacB	26,902	3.4	27,827.2 ± 0.5	+925.2 ^†^
			27,859.8 ± 0.7	+957.8 ^†^
		4.0	26,902.6 ± 0.2	+0.6 ^‡^
			26,930.4 ± 0.2	+28.4 ^‡, §^
		4.6	Phosphatidylethanolamines ^¶^	
Lip-PnrA	37,177	3.4	38,106.5 ± 0.9	+929.5 ^†^
		4.0	37,209.1 ± 2.7	+32.1 ^‡, §^
		4.7	Phosphatidylethanolamines ^¶^	

* Theoretical masses for lipidated proteins assuming that three palmitate residues are attached to the N-terminal cysteine of the protein sequences; ** Mean mass difference compared to the calculated theoretical mass for tri-palmitoylated proteins; ^†^ Posttranslationally unmodified protein fraction carrying the OspA signal sequence; ^‡^ Lipidated protein fraction; ^§^ Mass differences of 28 or 32 Da explainable by replacement of one C16 by a C18 acyl residue (Δmass = 28 Da) or by partial oxidations in the protein backbone at the cysteine and methionine positions (Δmass = 32 Da); ^¶^ Phospholipids derived from the cell membrane.

## Supplementary Materials

The following are available online at https://www.mdpi.com/2076-393X/8/2/310/s1. Figure S1: Protein sequences of recombinant lipidated and non-lipidated proteins; Figure S2: pETLip3 vector for N-terminal lipidation of recombinant proteins; Figure S3: Surface exposure of lipidated DacB and PnrA on recombinant *E. coli* ClearColi; Figure S4: Stimulation of HEK-Blue-hTLR2-KO-TLR1/6 cells with lipidated and non-lipidated DacB and PnrA; Figure S5: Electrospray mass spectrometry of recombinant lipidated and non-lipidated DacB; Figure S6: Electrospray mass spectrometry of recombinant lipidated and non-lipidated PnrA; Figure S7: IgG concentrations in pre-immune sera of intranasally and subcutaneously vaccinated mice; Figure S8: Antigen lipidation does not create new epitopes that are targeted by antibodies; Figure S9: Cytokine profile in the nasal tissue following intranasal vaccination in a mouse model of colonization; Figure S10: Impact of adjuvantation on the cytokine response in the nasal tissue or in in vitro restimulated splenocytes of immunized and pneumococcal-colonized mice; Figure S11: Cytokine profile following in vitro splenocyte restimulation of vaccinated mice; Figure S12: Correlation of nasopharyngeal bacterial loads with nasal antibody levels following subcutaneous immunization; Figure S13: Correlation of nasopharyngeal bacterial loads with systemic IgG subclass levels; Figure S14: Local IL-17A levels inversely correlate with reduced nasal bacterial loads.
