# Supplementary material for: Lipidation of Pneumococcal Antigens Leads to Improved Immunogenicity and Protection
Source: Vaccines (Basel). 2020 Jun 17;8(2):310. doi: 10.3390/vaccines8020310 (PMC7350230; doi:10.3390/vaccines8020310)
Supplement: Supplementary file 1 [file vaccines-08-00310-s001.pdf]

vaccines

Supplementary Information

Lipidation of pneumococcal antigens leads to improved immunogenicity and protection

Franziska Voß[1†], Lucille F. van Beek[2,3†], Dominik Schwudke[4,5,6], Thomas H. A. Ederveen[7], Fred J. van Opzeeland[2,3], Daniela Thalheim[1], Sidney Werner[1], Marien I. de Jonge[2,3‡] and Sven Hammerschmidt[1*‡]

1 Department of Molecular Genetics and Infection Biology, Interfaculty Institute of Genetics and Functional Genomics, Center for Functional Genomics of Microbes, University of Greifswald, 17489 Greifswald, Germany; franziska.voss@uni-greifswald.de (F.V.); da.thalheim@googlemail.com (D.T.); sag92@gmx.de (S.W.)
2 Section Pediatric Infectious Diseases, Laboratory of Medical Immunology, Radboud Institute for Molecular Life Sciences, Radboudumc, 6525 GA Nijmegen, The Netherlands; lucille.vanbeek@radboudumc.nl (L.F.v.B.); fred.vanopzeeland@radboudumc.nl (F.J.v.O.); marien.dejonge@radboudumc.nl (M.I.d.J.)
3 Radboud Center for Infectious Diseases, Radboudumc, 6525 GA Nijmegen, The Netherlands
4 Division of Bioanalytical Chemistry, Priority Area Infection, Research Center Borstel, Leibniz Center for Medicine and Bioscience, 23845 Borstel, Germany; dschwudke@fz-borstel.de
5 German Center for Infection Research (DZIF), 38124 Braunschweig, Germany
6 Airway Research Center North Member of the German Center for Lung Research (DZL), 22927 Großhansdorf, Germany
7 Center for Molecular and Biomolecular Informatics, Radboud Institute for Molecular Life Sciences, Radboud University Medical Center, 6525 GA Nijmegen, The Netherlands; tom.ederveen@radboudumc.nl
* Correspondence: sven.hammerschmidt@uni-greifswald.de; Phone: +49 3834 4205700, Fax: +49 3834 4205709
† These authors contributed equally to this work
‡ These senior authors contributed equally to this work.

Figure S1

(a) **Lip-DacB**

1 **9**

MKKYLLGIGLILALIA|**C**KQNVSSH**M**QEKTKNEDGETKTEQTAKADGTVGSKSQGAAQKKAEV
VNKGDYYSIQGKYDEIIVANKHYPLSKDYNPGENPTAKAELVKLIKAMQEAGFPISDHYSGFR
SYETQTKLYQDYVNQDGKAAADRYSARPGYSEHQTGLAFDVIGTDGDLVTEEKAAQWLLDHAA
DYGFVVRYLKGKEKETGYMAEEWHLRYVGKEAKEIAASGLSLEEYYGFEGGDYVDHHHHHH

(b) **DacB**

GSSHHHHHHMSGENLYFQGASQEKTKNEDGETKTEQTAKADGTVGSKSQGAAQKKAEVVNKGD
YYSIQGKYDEIIVANKHYPLSKDYNPGENPTAKAELVKLIKAMQEAGFPISDHYSGFRSYETQ
TKLYQDYVNQDGKAAADRYSARPGYSEHQTGLAFDVIGTDGDLVTEEKAAQWLLDHAADYGFV
VRYLKGKEKETGYMAEEWHLRYVGKEAKEIAASGLSLEEYYGFEGGDYVD

(c) **Lip-PnrA**

1 **9**

MKKYLLGIGLILALIA|**C**KQNVSSH**M**GNRSSRNAASSSDVKTKAAIVTDTGGVDDKSFNQSAW
EGLQAWGKEHNLSKDNGFTYFQSTSEADYANNLQQAAGSYNLIFGVGFALNNAVKDAAKEHTD
LNYVLIDDVIKDQKNVASVTFADNESGYLAGVAAAKTTKTKQVGFVGGIESEVISRFEAGFKA
GVASVDPSIKVQVDYAGSFGDAAKGKTIAAAQYAAGADIVYQVAGGTGAGVFAEAKSLNESRP
ENEKVWVIGVDRDQEAEGKYTSKDGKESNFVLVSTLKQVGTTVKDISNKAERGEFPGGQVIVY
SLKDKGVDLAVTNLSEEGKKAVEDAKAKILDGSVKVPEKHHHHHH

(d) **PnrA**

GSSHHHHHHMSGENLYFQGASGNRSSRNAASSSDVKTKAAIVTDTGGVDDKSFNQSAWEGLQA
WGKEHNLSKDNGFTYFQSTSEADYANNLQQAAGSYNLIFGVGFALNNAVKDAAKEHTDLNYVL
IDDVIKDQKNVASVTFADNESGYLAGVAAAKTTKTKQVGFVGGIESEVISRFEAGFKAGVASV
DPSIKVQVDYAGSFGDAAKGKTIAAAQYAAGADIVYQVAGGTGAGVFAEAKSLNESRPENEKV
WVIGVDRDQEAEGKYTSKDGKESNFVLVSTLKQVGTTVKDISNKAERGEFPGGQVIVYSLKDK
GVDLAVTNLSEEGKKAVEDAKAKILDGSVKVPEK

Figure S1. Protein sequences of recombinant lipidated and non-lipidated proteins. For the lipidated proteins (**a**,**c**), OspA signal peptide that is cleaved off after posttranslational modification is indicated by italic letters. Additional amino acids derived from OspA are underlined followed by the protein sequences for DacB or PnrA, respectively. The cysteine (position 1) and methionine (position 9) are possible oxidation sites. The C-terminal His$_6$-tag is highlighted by red letters. For the non-lipidated proteins (**b**,**d**), additional amino acids derived from the pTP1 expression vector are underlined followed by the protein sequences for DacB or PnrA, respectively [1,2]. The N-terminal His$_6$-tag is highlighted by red letters.

Figure S2

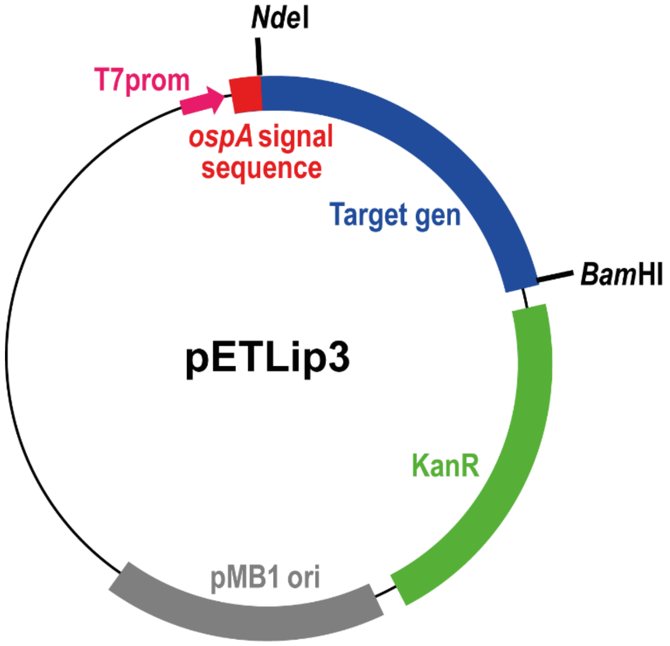

Figure S2. pETLip3 vector for N-terminal lipidation of recombinant proteins. For heterologous expression of lipidated proteins, the expression vector pETLip3 was used, which contains a T7 promotor followed by the signal sequence of *ospA* (red) from *Borrelia burgdorferi*. The selection of positive clones after transformation in *E. coli* was based on the kanamycin resistance gene *aphA3* (green).

Figure S3

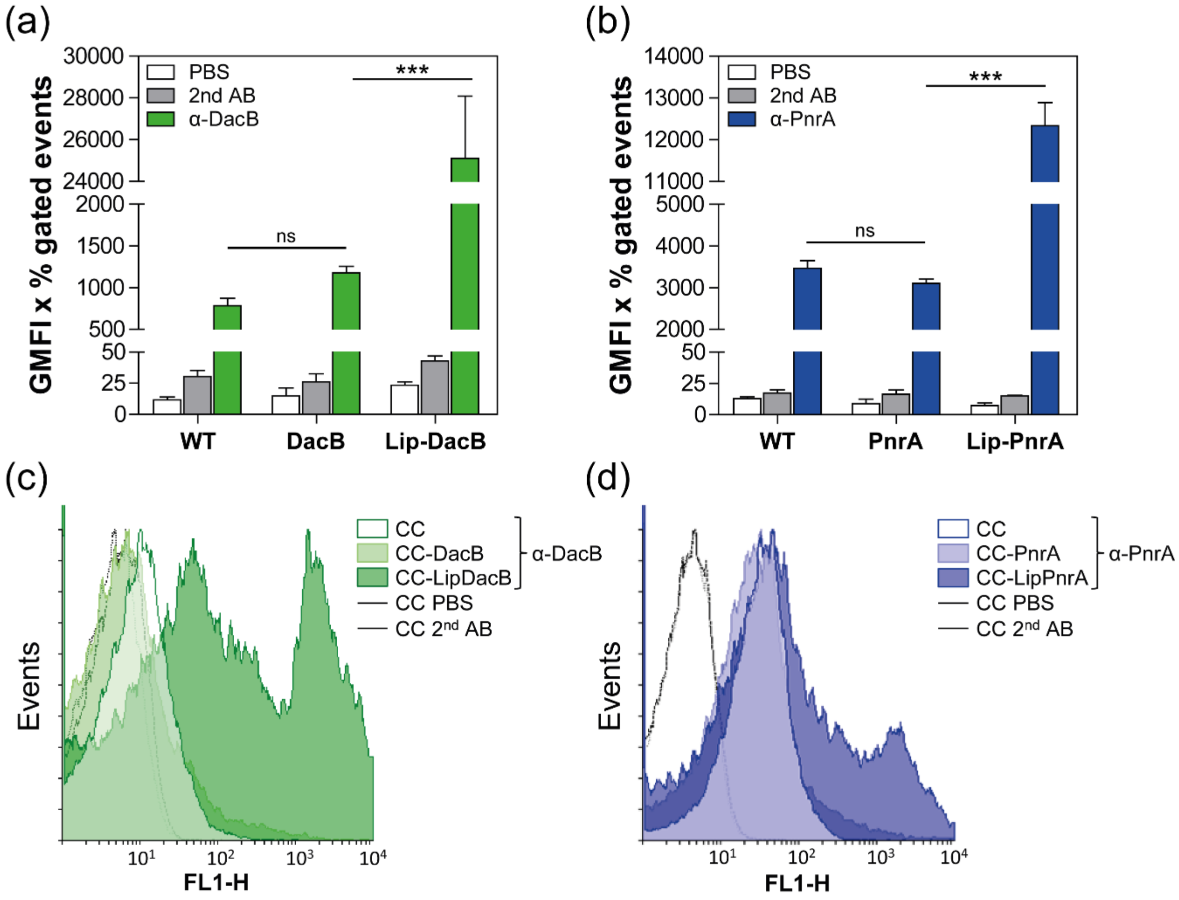

Figure S3. Surface exposure of lipidated DacB and PnrA on recombinant *E. coli* ClearColi. Following protein expression in recombinant *E. coli* ClearColi® (CC) strains, surface exposure of lipidated and non-lipidated DacB (**a**,**c**) and PnrA (**b**,**d**) was determined by flow cytometry with lipoprotein-specific antibodies. Wild-type and recombinant *E. coli* ClearColi® incubated with PBS or secondary antibody only were used as negative control. (a,b) Geometric mean fluorescence intensity (GMFI) x % gated events of four independent experiments is shown with error bars corresponding to SEM. Statistical significance was determined using one-way ANOVA analysis of variance with Bonferroni's post-test. ***, $p < 0.001$; ns, not significant. (c,d) Representative histograms of four independent experiments.

Figure S4

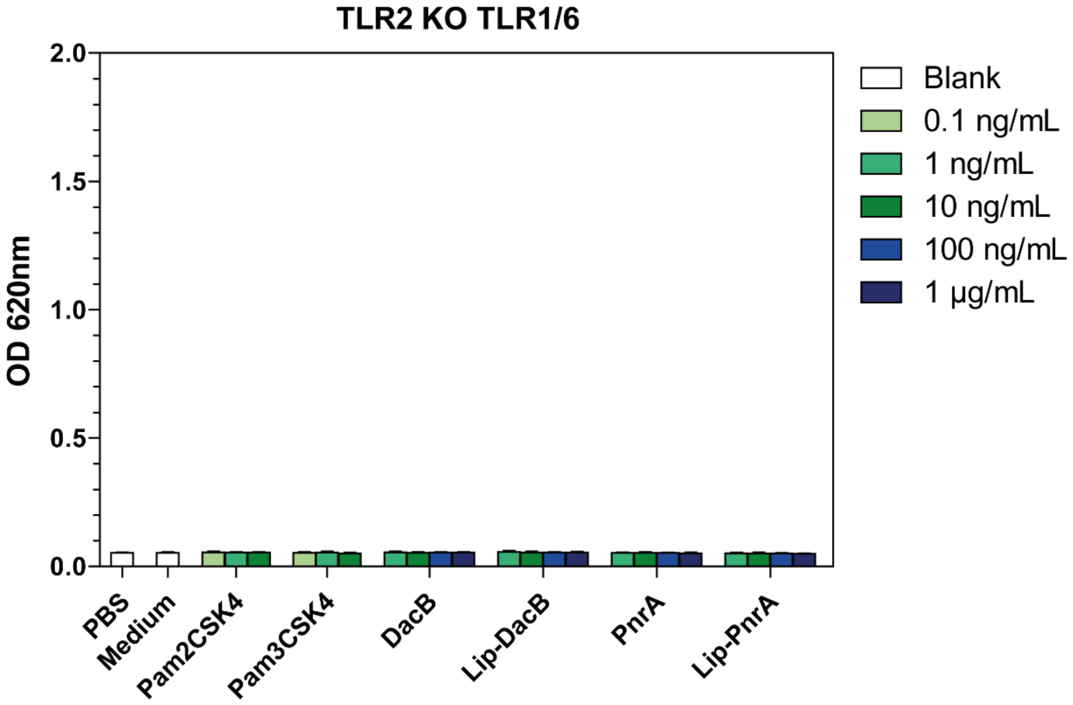

Figure S4. Stimulation of HEK-Blue-hTLR2-KO-TLR1/6 cells with lipidated and non-lipidated DacB and PnrA. HEK-Blue-hTLR2-KO-TLR1/6 cells were stimulated with increasing doses of Pam2CSK4, Pam3CSK4, lipidated and non-lipidated proteins DacB or PnrA. Following stimulation in HEK-Blue detection medium over 15 h, the OD at 620 nm was measured. Results of three independent experiments are shown with error bars corresponding to SD.

Figure S5

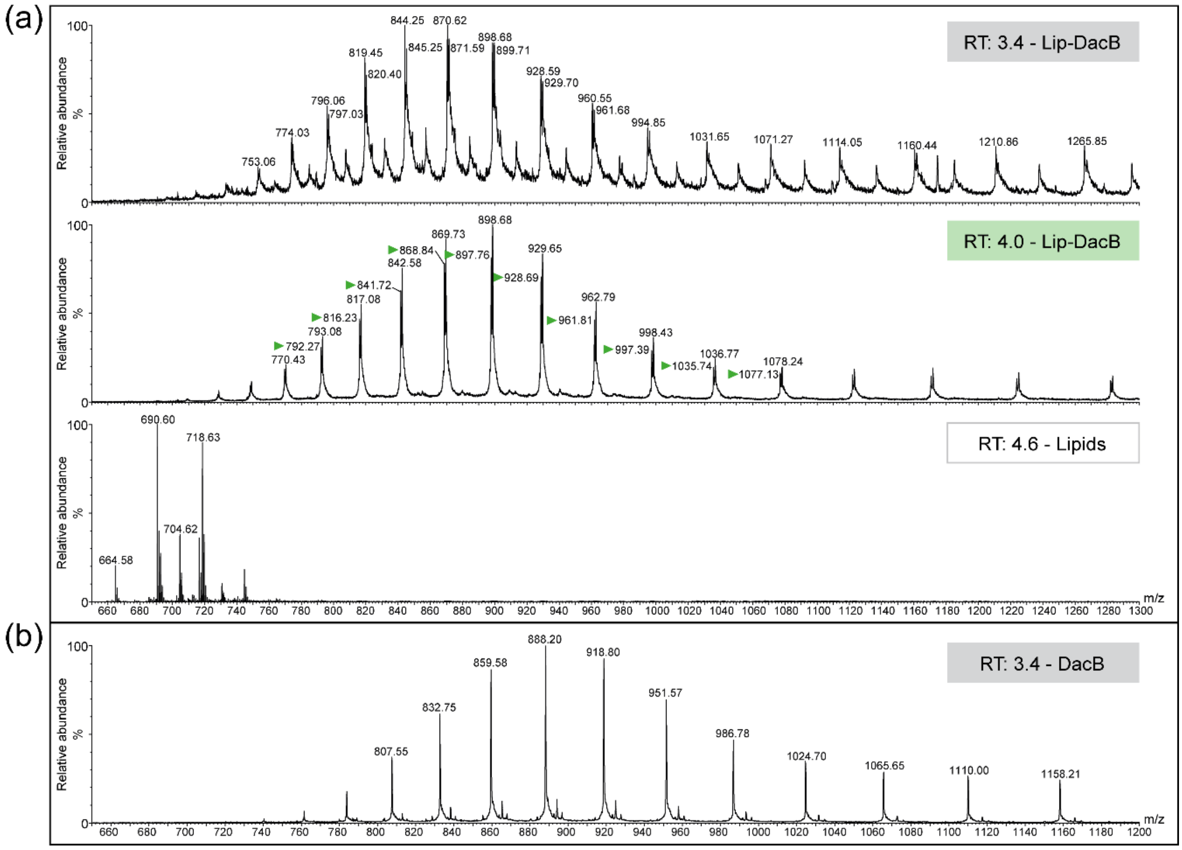

Figure S5. Electrospray mass spectrometry of recombinant lipidated and non-lipidated DacB. Lipidated (**a**) and non-lipidated (**b**) DacB were analyzed by LC–MS. HPLC elution profiles of lipidated DacB were composed of three major components (a): i) posttranslationally unmodified protein (RT: 3.4 min), ii) lipidated protein (RT: 4.0 min), and iii) phospholipids (RT: 4.6 min) from the cell membrane (Table 2, Figure 1c in the main manuscript). Expected mass of lipidated DacB is 26,902 Da, assuming three palmitate residues attached to the N-terminal cysteine of the protein sequence. The protein species at RT 3.4 min revealed 925 Da or 958 Da higher masses compared to the expected mass of palmitoylation and thus probably represents posttranslationally unmodified protein still carrying the OspA signal sequence. The spectrum at RT 4.0 min revealed heterogeneity of the recombinant lipidated DacB preparation. Nevertheless, a mass of 26,902.6 ± 0.6 Da (▶) was measured for one of the two major components, which corresponds to the mass of expected palmitoylation. The other series had a measured mass of 26,930.4 ± 0.2 Da, which might be explained by partial oxidations in the protein backbone at the cysteine and methionine (Δmass = 32 Da) or by replacement of one C16 by a C18 acyl residue (Δmass = 28 Da). The masses detected at RT 4.6 min correspond to phospholipids derived from the cell membrane. (b) For non-lipidated DacB, a mass of 26,615.1 ± 2 Da was measured at RT 3.4 min, which corresponds to the expected mass of 26,614 Da. RT: retention time in HPLC

Figure S6

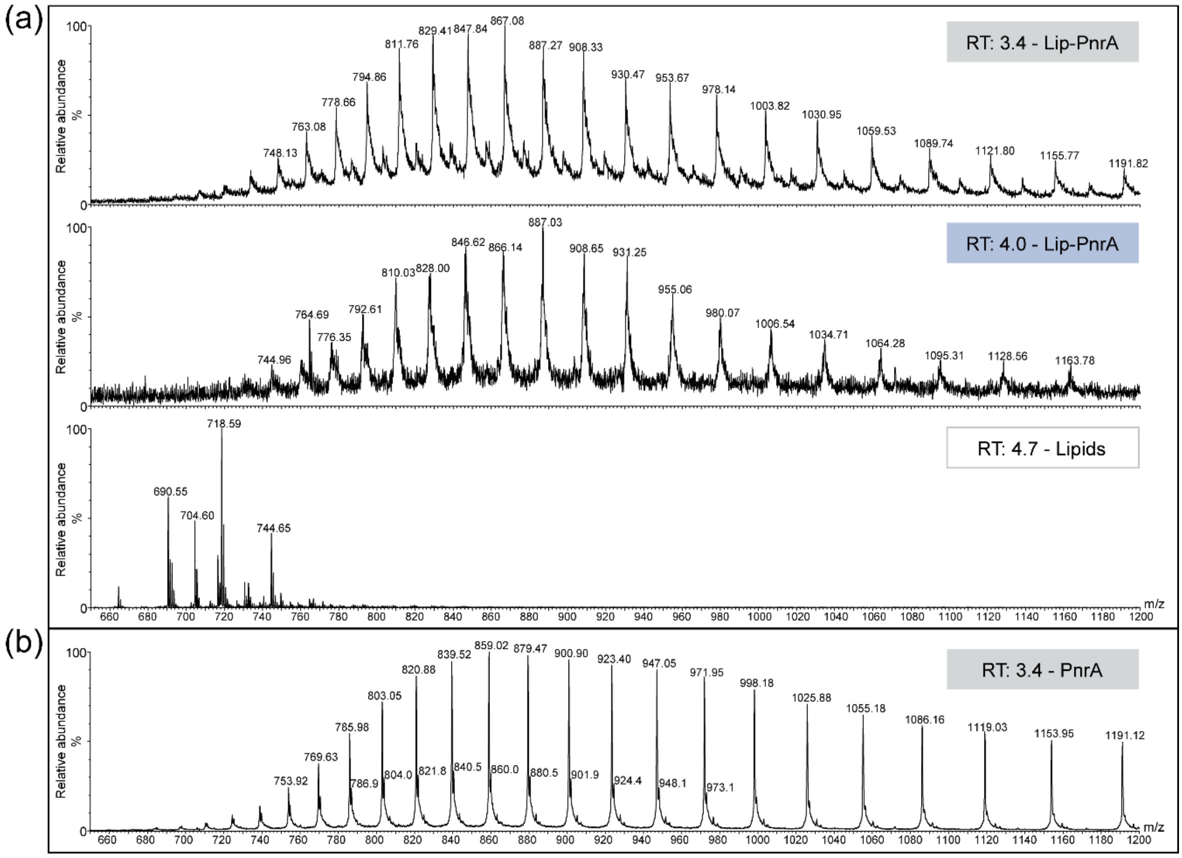

Figure S6. Electrospray mass spectrometry of recombinant lipidated and non-lipidated PnrA. Lipidated (**a**) and non-lipidated (**b**) PnrA were analyzed by LC–MS. HPLC elution profiles of lipidated PnrA were composed of three major components (a): i) posttranslationally unmodified protein (RT: 3.4 min), ii) lipidated protein (RT: 4.0 min), and iii) phospholipids (RT: 4.7 min) from the cell membrane (Table 2, Figure 1d in the main manuscript). Expected mass of lipidated PnrA is 37,177 Da, assuming three palmitate residues attached to the N-terminal cysteine of the protein sequence. The protein species at RT 3.4 min revealed 929 Da higher masses compared to the expected mass of palmitoylation and thus probably represents posttranslationally unmodified protein still carrying the OspA signal sequence. The spectrum at RT 4.0 min revealed a mass of 37,209.1 ± 2.7 Da representing the lipidated PnrA. The mass difference of around 30 Da might be explained by partial oxidations in the protein backbone at the cysteine and methionine (Δmass = 32 Da) or by replacement of one C16 by a C18 acyl residue (Δmass = 28 Da). The masses detected at RT 4.7 min correspond to phospholipids derived from the cell membrane. (b) For non-lipidated PnrA, a mass of 36,895 ± 1.2 Da was measured at RT 3.4 min, which is close to the expected mass of 36,890 Da. An additional minor component of unknown origin was detected. RT: retention time in HPLC

Figure S7

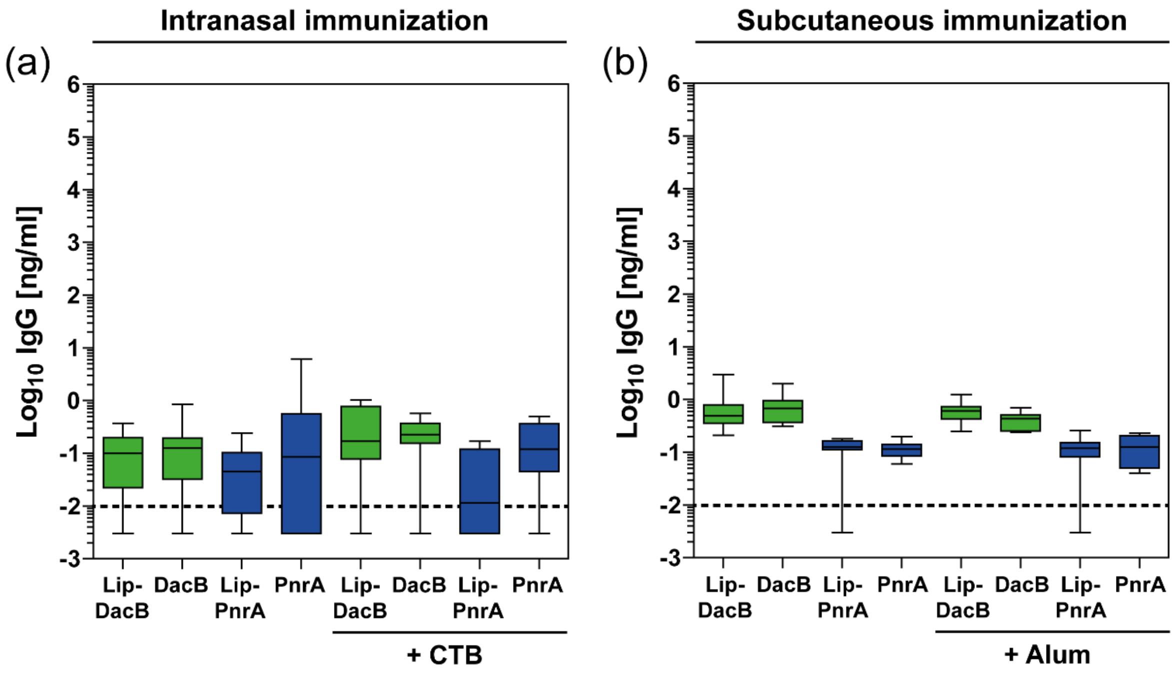

Figure S7. IgG concentrations in pre-immune sera of intranasally and subcutaneously vaccinated mice. Before the first intranasal (**a**) or subcutaneous (**b**) vaccination of C57BL/6 mice (*n* = 8/group), sera were collected and monitored for their antigen-specific IgG concentrations using ELISA. Box plots represent group median (horizontal line), first and third quartiles (box), and the range of data (whiskers). The dashed line indicates the lower limit of detection.

Figure S8

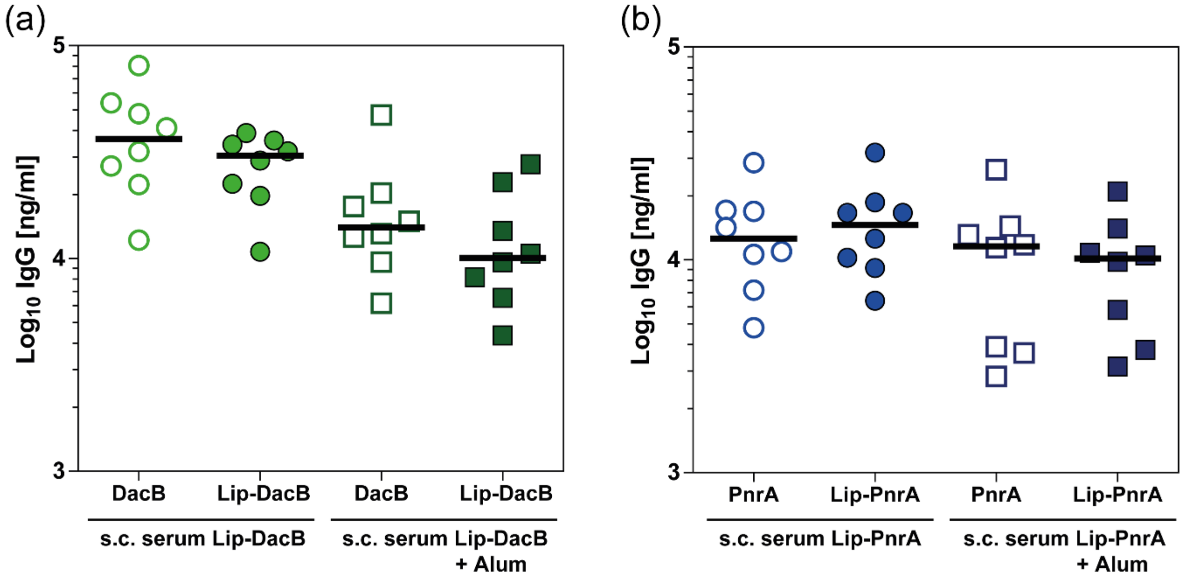

Figure S8. Antigen lipidation does not create new epitopes that are targeted by antibodies. Antigen-specific IgG concentrations (Log_{10} ng/mL) in post-immune sera of C57BL/6 mice (*n* = 8/group) receiving three subcutaneous vaccinations with 5 µg of Lip-DacB (**a**) or Lip-PnrA (**b**) with or without additional adjuvant were monitored by ELISA. For immobilization, equimolar amounts of either non-lipidated or lipidated protein (3 pmol) was used to compare the IgG levels and examine the impact of lipidation on the magnitude of the antibody response. Symbols represent individual mice with solid lines representing the group median.

Figure S9

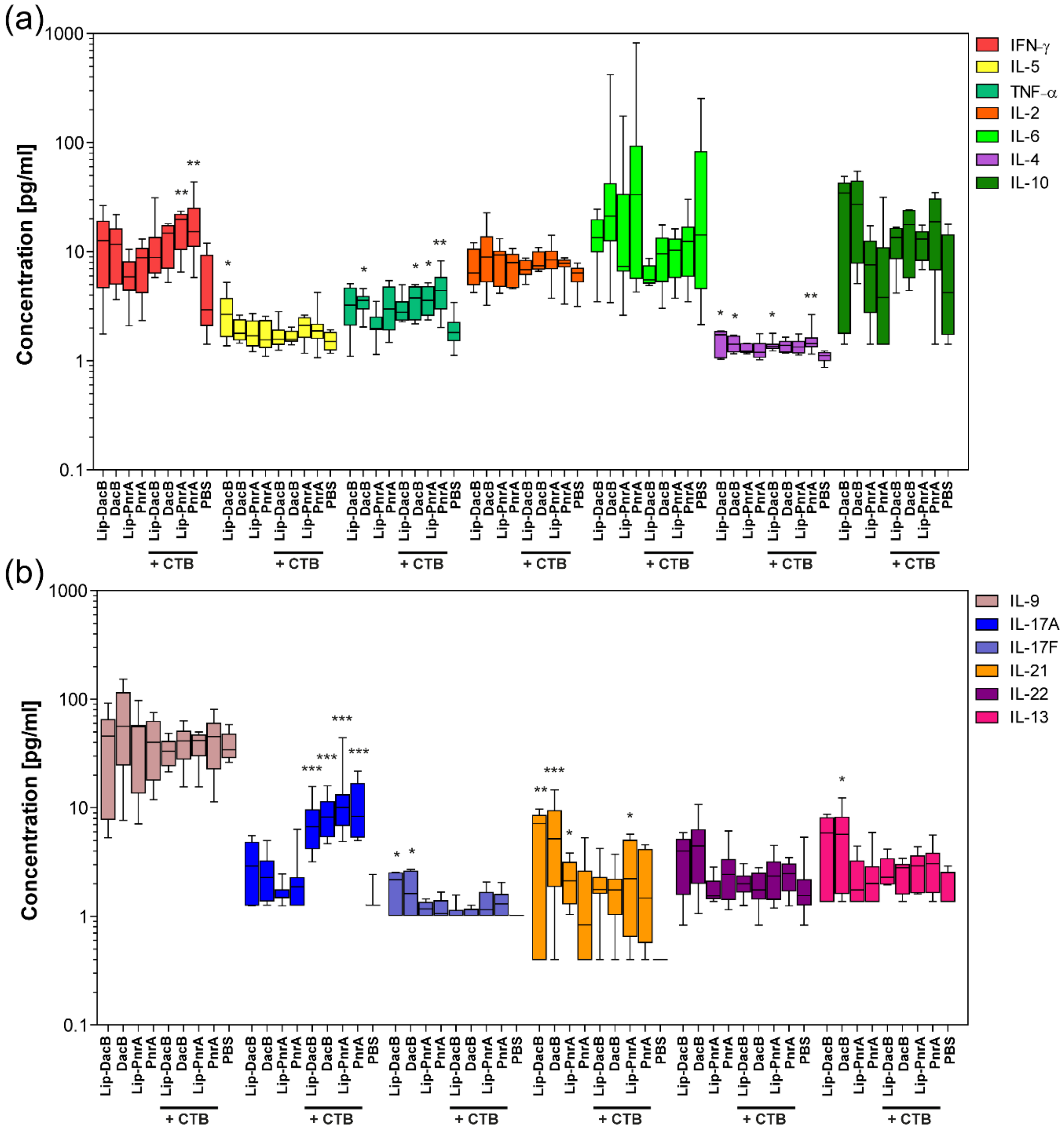

Figure S9. Cytokine profile in the nasal tissue following intranasal vaccination in a mouse model of colonization. Nasal tissue was analyzed of C57BL/6 mice (n = 8/group) that were intranasally immunized followed by an intranasal infection with 10^6 CFU *S. pneumoniae* three days prior to tissue collection. Cytokine levels (lower limits of detection provided in parentheses) were determined using a flow cytometric bead assay to detect IFN-γ (LOD: 1.11 pg/mL), IL-5 (0.73 pg/mL), TNF-α (0.69 pg/mL), IL-2 (0.53 pg/mL), IL-6 (0.74 pg/mL), IL-4 (0.48 pg/mL), IL-10 (1.42 pg/mL), IL-9 (1.36 pg/mL), IL-17A (1.27 pg/mL), IL-17F (1.02 pg/mL), IL-21 (0.40 pg/mL), IL-22 (0.83 pg/mL), and IL-13 (1.37 pg/mL). Box plots represent group median (horizontal line), first and third quartiles (box), and the range of data (whiskers). Statistical significance was determined using a Kruskal Wallis test accompanied by Dunn's multiple comparison post-test, with all conditions compared to PBS-treated mice. *, $p < 0.05$; **, $p < 0.01$; ***, $p < 0.001$. LOD – lower limit of detection

Figure S10

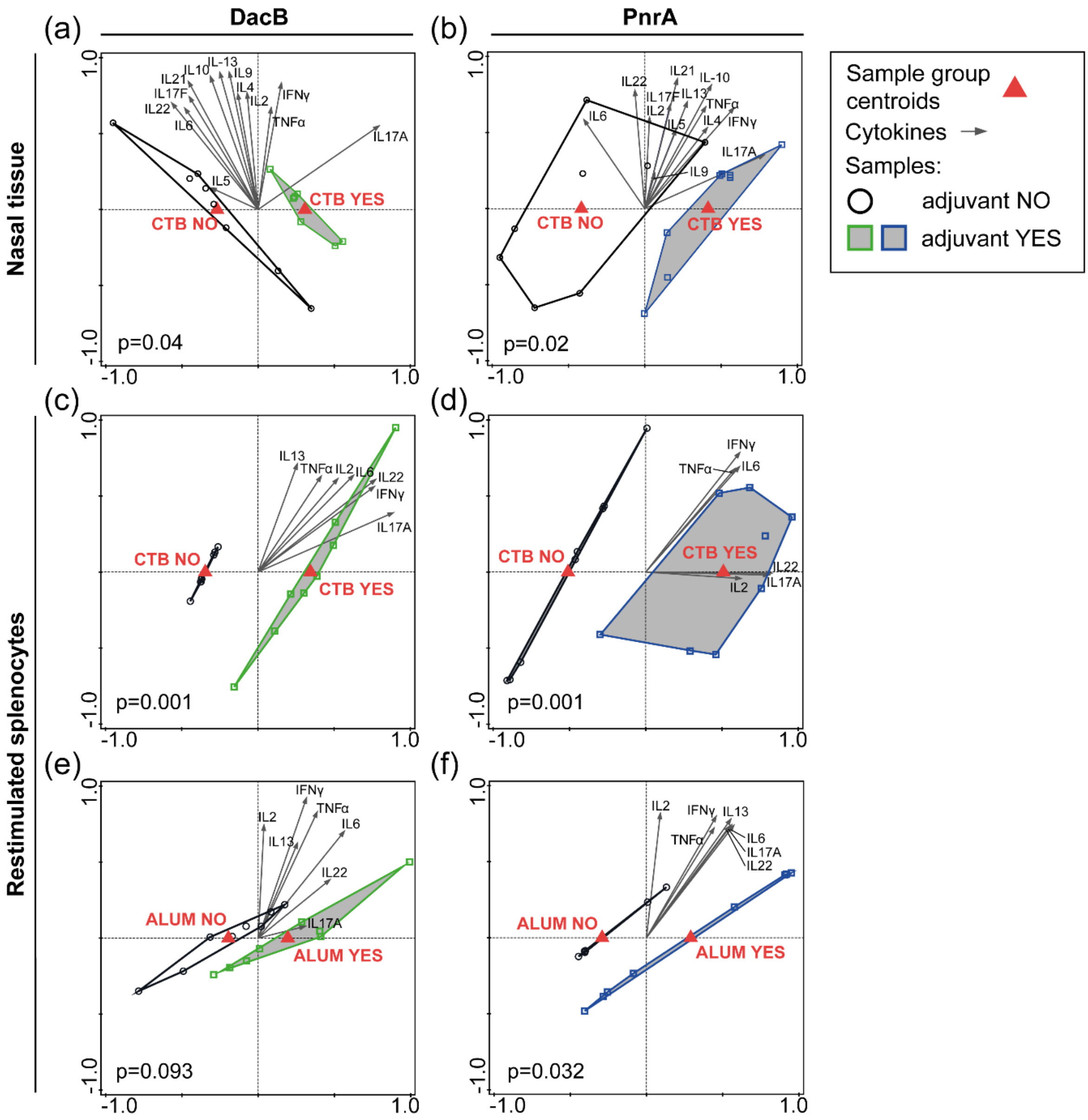

Figure S10. Impact of adjuvantation on the cytokine response in the nasal tissue or in *in vitro* restimulated splenocytes of immunized and pneumococcal colonized mice. Nasal tissue was analyzed of C57BL/6 mice (*n* = 8/group) that were intranasally immunized with lipidated or non-lipidated DacB (**a**) or PnrA (**b**) followed by an intranasal infection with *S. pneumoniae* three days prior to collection. Cytokine levels in tissue homogenate were used for RDA to determine local CTB-specific cytokine signatures for both proteins. Splenocytes were obtained from C57BL/6 mice (*n* = 4/group) that were intranasally (**c**,**d**) or subcutaneously (**e**,**f**) immunized followed by the pneumococcal infection. Cells were restimulated *in vitro* with lipidated proteins, matching the vaccination background, for 72 h. The cytokine levels were assessed and used for RDA irrespective of lipidation to evaluate systemic adjuvant-specific cytokine signatures for DacB (c,e) and PnrA (d,f). Adjuvant YES refers to mice vaccinated with adjuvanted proteins; and Adjuvant NO refers to mice vaccinated with non-adjuvanted proteins. Arrows indicate individual cytokines, red triangles the sample group centroids, and squares/circles represent individual mice. *p*-values are shown, $p < 0.05$ is considered significant.

Figure S11

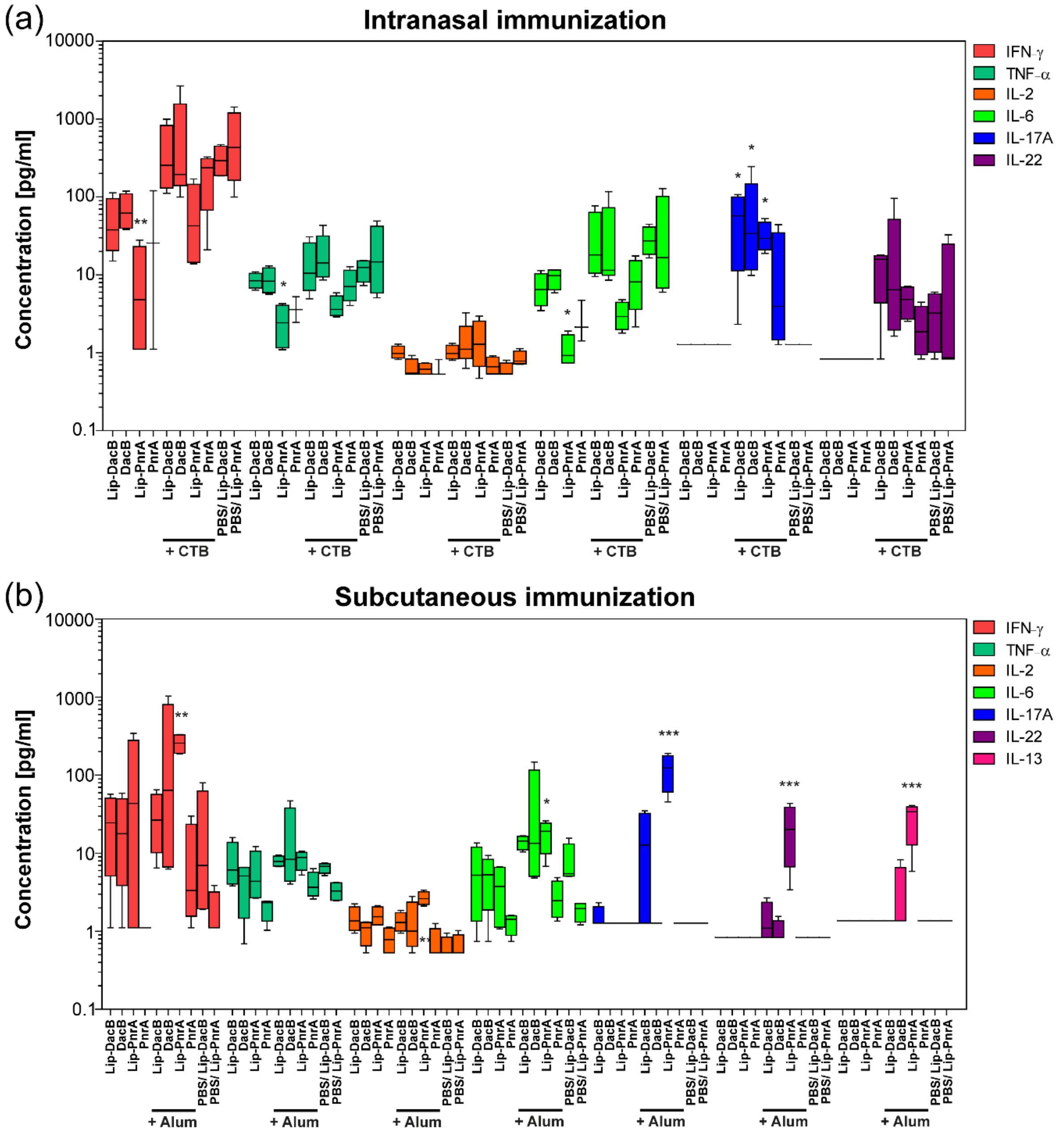

Figure S11. Cytokine profile following *in vitro* splenocyte restimulation of vaccinated mice. Cytokines produced upon *in vitro* restimulation of splenocytes isolated from C57BL/6 mice (n = 4/group) that were intranasally (**a**) or subcutaneously (**b**) immunized followed by an intranasal infection with 1×10^6 CFU *S. pneumoniae* three days prior to collection. Cytokine levels (lower limits of detection provided in parentheses) were determined using a flow cytometric bead assay to detect IFN-γ, IL-5, TNF-α, IL-2, IL-6, IL-4, IL-10, IL-9, IL-17A, IL-17F, IL-21, IL-22, and IL-13, with only cytokines detected included in the graph. The lower limits of detection are indicated in Figure S9. Spleen cells from PBS-treated mice were stimulated with Lip-DacB or Lip-PnrA. Cultivation of spleen cells without stimulant were used as a negative control. For normalization of cytokine levels, baseline values of the negative control were subtracted from the concentrations measured with the respective lipidated proteins. Box plots represent group median (horizontal line), first and third quartiles (box), and the range of data (whiskers). Statistical significance was determined using a Kruskal Wallis test accompanied by Dunn's multiple comparison post-test, with conditions compared to spleens from PBS-treated mice stimulated with the respective lipidated protein. *, $p < 0.05$; **, $p < 0.01$; ***, $p < 0.001$.

Figure S12

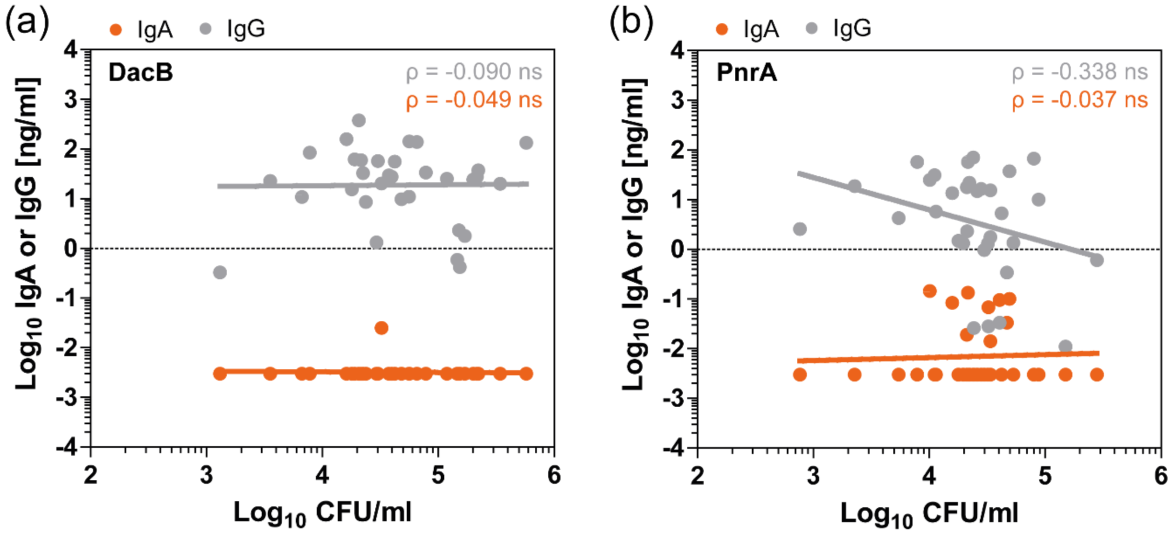

Figure S12. Correlation of nasopharyngeal bacterial loads with nasal antibody levels following subcutaneous immunization. Paired analysis of CFU counts and nasopharyngeal IgA and IgG levels of subcutaneously DacB- (**a**) or PnrA-immunized (**b**) mice with data of vaccinations with lipidated or non-lipidated DacB or PnrA combined, respectively. Symbols represent individual mice (n = 8/group). Analysis was performed on log-transformed data. Spearman's coefficients (ϱ) are shown (ns – not significant).

Figure S13

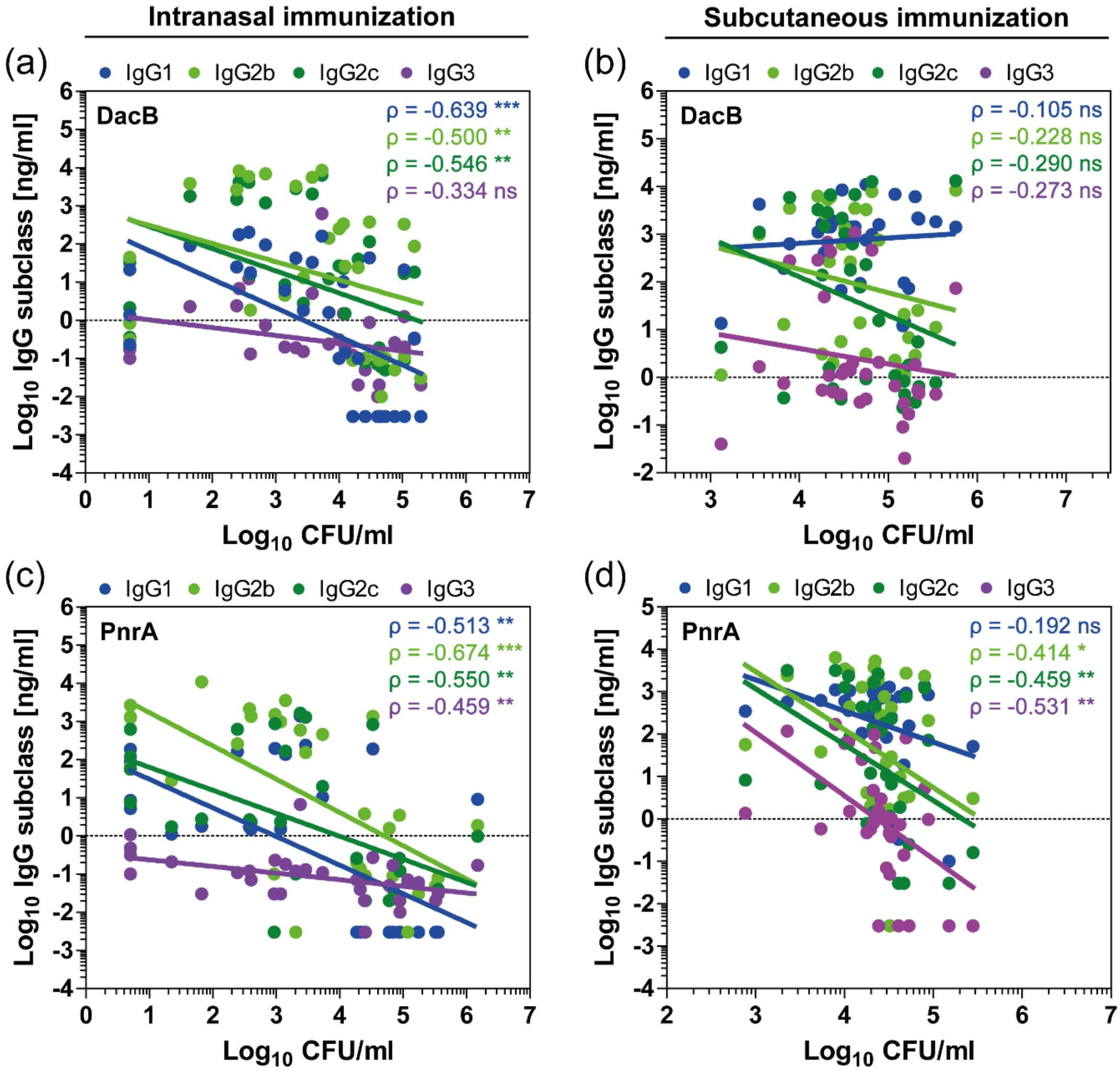

Figure S13. Correlation of nasopharyngeal bacterial loads with systemic IgG subclass levels. Paired analysis of systemic IgG subclass levels and CFU counts of intranasally (**a**,**c**) or subcutaneously (**b**,**d**) DacB- (a,b) or PnrA-immunized (c,d) mice with data of vaccinations with lipidated or non-lipidated DacB or PnrA combined, respectively. Symbols represent individual mice (n = 8/group). Analysis was performed on log-transformed data. Spearman's coefficient (ϱ) and p-value are shown (*, $p < 0.05$; **, $p < 0.01$; ***, $p < 0.001$). The coefficients of determination (R^2) of the linear regression are the following: Intranasal immunization – DacB (**a**): 0.302 (IgG1), 0.103 (IgG2b), 0.175 (IgG2c), 0.070 (IgG3); PnrA (**c**): 0.320 (IgG1), 0.433 (IgG2b), 0.289 (IgG2c), 0.191 (IgG3); Subcutaneous immunization – DacB (**b**): 0.006 (IgG1), 0.039 (IgG2b), 0.076 (IgG2c), 0.021 (IgG3); PnrA (**d**): 0.100 (IgG1), 0.111 (IgG2b), 0.133 (IgG2c), 0.246 (IgG3)

Figure S14

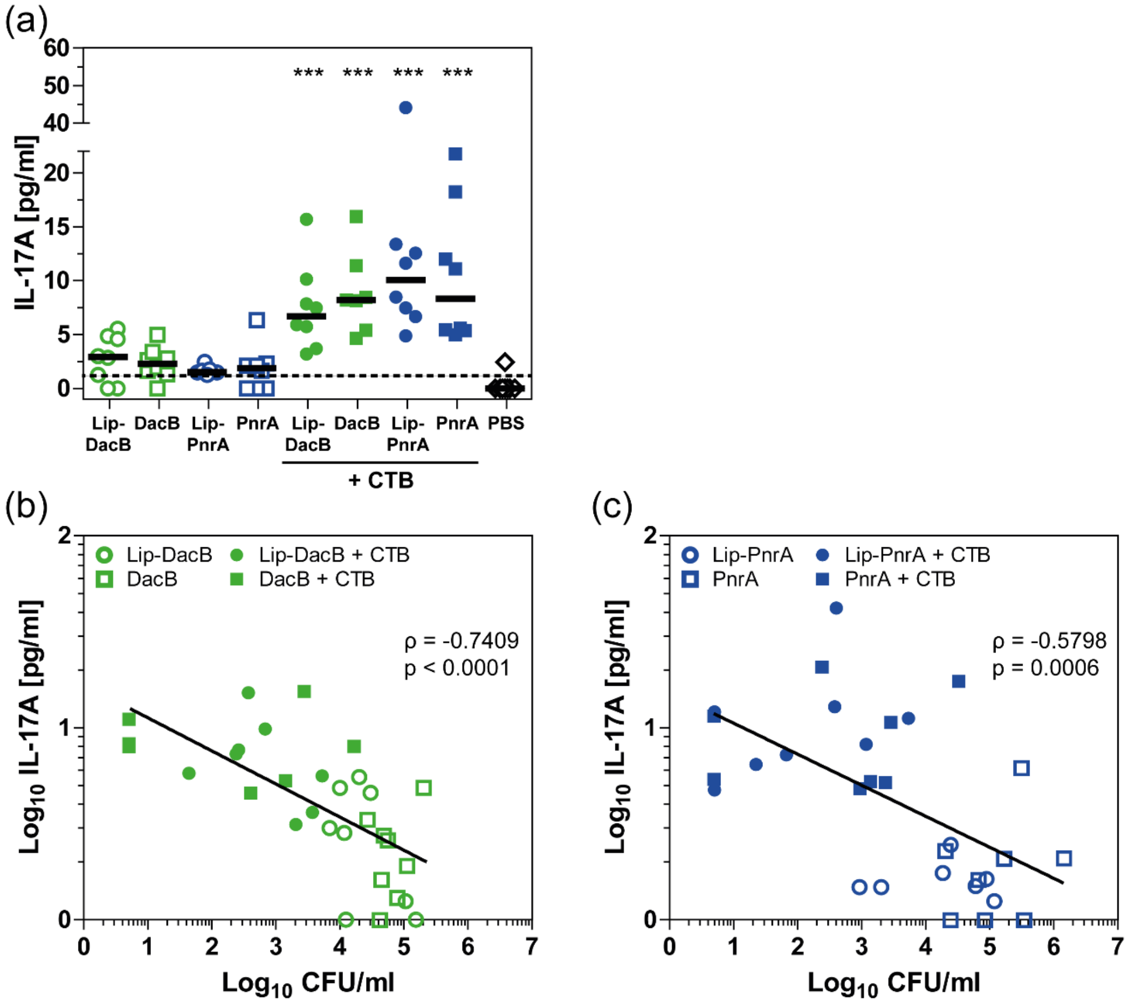

Figure S14. Local IL-17A levels inversely correlate with reduced nasal bacterial loads. Nasopharyngeal IL-17A levels (**a**) of intranasally immunized C57BL/6 mice (n = 8/group) three days post intranasal infection with *S. pneumoniae.* Symbols represent individual mice, solid lines the group median, and the dashed line indicates the lower limit of detection. Data was statistically analyzed by a Kruskal Wallis test accompanied by Dunn's multiple comparison post-test, with all conditions compared to PBS-treated (***, $p < 0.001$). Paired analysis of CFU counts and nasopharyngeal IL-17A levels of DacB- (**b**) or PnrA-immunized (**c**) mice. Symbols represent individual mice (n = 8/group). Analysis was performed on log-transformed data. Spearman's coefficient (ϱ) and p-value are shown. The coefficients of determination (R^2) of the linear regressions for DacB and PnrA are 0.4505 and 0.3267, respectively.

References

1. Abdullah, M.R.; Gutierrez-Fernandez, J.; Pribyl, T.; Gisch, N.; Saleh, M.; Rohde, M.; Petruschka, L.; Burchhardt, G.; Schwudke, D.; Hermoso, J.A., et al. Structure of the pneumococcal L,D-carboxypeptidase DacB and pathophysiological effects of disabled cell wall hydrolases DacA and DacB. *Mol Microbiol* **2014**, *93*, 1183-1206, doi:10.1111/mmi.12729.
2. Voss, F.; Kohler, T.P.; Meyer, T.; Abdullah, M.R.; van Opzeeland, F.J.; Saleh, M.; Michalik, S.; van Selm, S.; Schmidt, F.; de Jonge, M.I., et al. Intranasal Vaccination With Lipoproteins Confers Protection Against Pneumococcal Colonisation. *Front Immunol* **2018**, *9*, 2405, doi:10.3389/fimmu.2018.02405.
